# Generation of a double binary transgenic zebrafish model to study myeloid gene regulation in response to oncogene activation in melanocytes

**DOI:** 10.1242/dmm.030056

**Published:** 2018-04-06

**Authors:** Amy Kenyon, Daria Gavriouchkina, Jernej Zorman, Vanessa Chong-Morrison, Giorgio Napolitani, Vincenzo Cerundolo, Tatjana Sauka-Spengler

**Affiliations:** 1 University of Oxford, Weatherall Institute of Molecular Medicine, Radcliffe Department of Medicine, Oxford OX3 9DS, United Kingdom; 2 University of Oxford, Weatherall Institute of Molecular Medicine, MRC Human Immunology Unit, Radcliffe Department of Medicine, Oxford OX3 9DS, United Kingdom

**Keywords:** Biotagging, Macrophage, Melanocyte, Neutrophil, Oncogene, Zebrafish

## Abstract

A complex network of inflammatory genes is closely linked to somatic cell transformation and malignant disease. Immune cells and their associated molecules are responsible for detecting and eliminating cancer cells as they establish themselves as the precursors of a tumour. By the time a patient has a detectable solid tumour, cancer cells have escaped the initial immune response mechanisms. Here, we describe the development of a double binary zebrafish model that enables regulatory programming of the myeloid cells as they respond to oncogene-activated melanocytes to be explored, focussing on the initial phase when cells become the precursors of cancer. A hormone-inducible binary system allows for temporal control of expression of different Ras oncogenes (*NRas*^Q61K^, *HRas*^G12V^ and *KRas*^G12V^) in melanocytes, leading to proliferation and changes in morphology of the melanocytes. This model was coupled to binary cell-specific biotagging models allowing *in vivo* biotinylation and subsequent isolation of macrophage or neutrophil nuclei for regulatory profiling of their active transcriptomes. Nuclear transcriptional profiling of neutrophils, performed as they respond to the earliest precursors of melanoma *in vivo*, revealed an intricate landscape of regulatory factors that may promote progression to melanoma, including Serpinb1l4, Fgf1, Fgf6, Cathepsin H, Galectin 1 and Galectin 3. The model presented here provides a powerful platform to study the myeloid response to the earliest precursors of melanoma.

## Abstract (precis)

**Summary:** The authors present an innovative double binary zebrafish model for exploring regulatory mechanisms that govern the myeloid response at the onset of melanoma.

## INTRODUCTION

Immune cells and their associated molecules are responsible for detecting and eliminating cancer cells and their precursors at early stages of cancer development (Williams et al., 2016; de Visser et al., 2006). A complex network of inflammatory genes is closely linked to somatic cell transformation and malignant disease. Importantly, analysis of the initial response of immune cells to cancer might lead to the discovery of targets for both prevention and treatment. As with pathogens, cancer cells can evade the immune system and as such tumour growth and ultimately the emergence of clinically detectable cancer is largely dependent on the capacity of cancerous cells to evade and manipulate the immune response (Blair and Cook, 2008). Myeloid cells are equipped with sensors for damage and inflammation as well as subsequent effector mechanisms for resolving inflammation (Feng et al., 2010). However, transformed cells are able to shape the nature of the myeloid cells and evoke an immunosuppressive response, enabling progression of the disease (Engblom et al., 2016). Further studies are needed to understand the different inflammatory signalling pathways in tumour initiation and progression, and how they may be modulated for immunotherapy. Because we cannot predict when and where transformed cells may arise in the human host, little is known about the sterile inflammatory signalling pathways that closely follow somatic cell transformation (Feng et al., 2012).

The zebrafish is an accepted model in cancer research, with many aspects of carcinogenesis being conserved between teleosts and humans (White et al., 2013; Stern and Zon, 2003). Unlike mammalian models, the zebrafish is easily amenable to techniques that allow the study of both cancer initiation and progression (Feng et al., 2010). Zebrafish are particularly useful in studying melanoma because their melanocytes are externally visible and large single cells can be directly visualised live (Ceol et al., 2008).

Previous transgenic zebrafish melanoma models used oncogenic *BRAF*^V600E^ (Patton et al., 2005), *NRas*^Q61K^ (Dovey et al., 2009) and *HRas*^G12V^ (Michailidou et al., 2009; Anelli et al., 2009) under the melanocyte-specific microphthalmia-associated transcription factor a (*mitfa*) promoter, coupled to an additional mutation in the tumour suppressor gene *p53*. In the *mitfa*-driven *HRas*^G12V^ transgenic line, melanoma development is rare but secondary mutations in genes involved in the PI3K signalling pathway contribute to its occurrence (Patton et al., 2005; Dovey et al., 2009; Anelli et al., 2009). Santoriello et al. subsequently developed a conditional transgenic zebrafish line that uses the Gal4 system and the melanocyte-specific *kita* promoter to drive *HRas*^G12V^ expression (Vallone et al., 2007; Santoriello et al., 2010). kita:HRas^G12V^ induced early-onset melanoma without additional mutations in tumour suppressor genes. However, some reports suggest that high levels of *Gal4* expression can be toxic and may result in developmental defects (Emelyanov and Parinov, 2008). Moreover, upstream activating sequences (UAS) have been shown to be susceptible to DNA methylation leading to transcriptional silencing of the transgene, which is minimal in the first generation but exacerbated upon propagation through later generations (Goll et al., 2009; Akitake et al., 2011).

The study of the myeloid response to the earliest precursors of melanoma would require temporal control of carcinogenesis. The current zebrafish models for benign naevus and cutaneous melanoma rely on the targeted expression of a human oncogene in melanocytes, limiting the ability to control melanoma initiation. Additionally, constitutive expression of the oncogene renders maintenance of stable transgenic lines difficult because these models may develop severe tumours before the fish reach reproductive age (Nguyen et al., 2012).

To overcome these limitations, we set out to develop an inducible system of melanoma initiation. Here, we report the generation of a new inducible zebrafish model, specifically designed to study the onset of melanoma using the mifepristone-inducible LexPR system developed by Emelyanov and Parinov (Emelyanov and Parinov, 2008). Additionally, we have developed an innovative transgenic zebrafish model system for *in vivo* biotinylation and subsequent isolation of neutrophil (previously described in Kenyon et al., 2017) and macrophage nuclei based on the biotagging method (Trinh et al., 2017). Using this approach, it is possible to perform genome-wide analysis of the active nuclear transcriptomes of neutrophils and macrophages. By combining the biotagging approach that enables isolation and analysis of either neutrophil or macrophage nuclei with the inducible model for melanoma initiation, we have created a powerful double binary system for regulatory profiling of myeloid cells that respond to the precursors of melanoma. In a proof-of-concept analysis, we reveal a number of interesting factors upregulated by neutrophils responding to the oncogene-activated melanocytes that may promote melanoma progression.

## RESULTS

### Generation of a binary inducible melanoma model

**Fig. 1.**
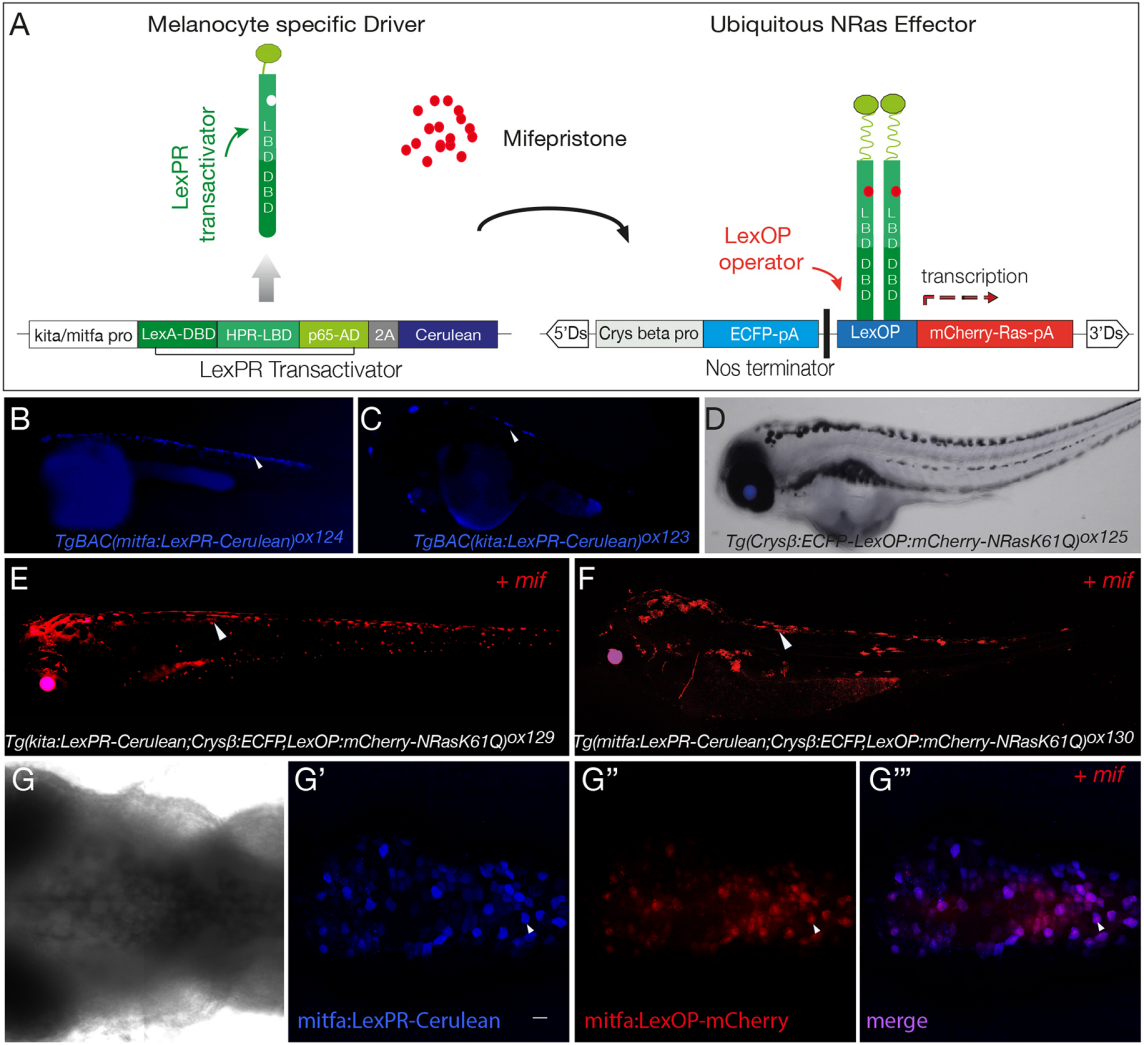
**LexPR mifepristone-inducible model for melanocyte oncogenic activation.** (A) Diagram of LexPR/LexOP inducible system. The LexPR driver cassette consists of LexPR transactivator and a Cerulean reporter under the control of melanocyte-specific promoters, *kita* or *mitfa*. The effector cassette contains the Lex operator sequence (LexOP) fused to the mCherry–Ras-oncogene. mCherry-Ras fusion is transcribed in trans only in the presence of mifepristone, when the LexPR transactivator-mifepristone complex binds to the LexOP sequence upstream of the oncogene expression cassette. (B,C) *mitfa:LexPR-Cerulean* [*TgBAC(mitfa:LexPR-Cerulean)^ox124^*] and *kita:LexPR-Cerulean* [*TgBAC(kita:LexPR-Cerulean)^ox123^*] transactivator driver lines were characterised by expression of LexPR transactivator and its cognate fluorescent protein Cerulean specifically in melanocytes. (D) *LexOP:mCherry-NRas* effector line [*Tg(Crysβ:ECFP-LexOP:mCherry-NRas^*Q61K*^)^ox125^*] shows ECFP expression in the eye, but no oncogene transcription. (E,F) The melanocyte-specific expression of mCherry-NRas in transgenic larvae harbouring both a transactivation driver and an oncogene effector allele [*Tg(kita/mitfa:LexPR-Cerulean;LexOP:mCherry-NRas^*Q61K*^)^ox129/ox130^*] is activated by addition of 1 μM mifepristone to the embryo rearing solution. (G-G‴). Confocal *z*-stack projection of the dorsal view of the melanocytes in the hindbrain region of a *Tg(mitfa:LexPR-Cerulean;LexOP:mCherry-NRas^*Q61K*^)^ox130^* transgenic larvae shows brightfield view (G). LexPR-Cerulean fusion (blue) (G′) and mifepristone-dependent activation of LexOP:mCherry-NRas^Q61K^ fusion (red) (G″) and the overlap (purple) (G‴). Larvae were partially bleached to attenuate the pigment and the mCherry signal was amplified with an anti-Cherry antibody. White arrowheads show Cerulean and mCherry signal localised to the melanocytes. Scale bar: 25 μm.

To generate an inducible model for the initiation of melanoma tumorigenesis, we adapted a hormone-inducible binary system for targeted gene expression in zebrafish, previously used to drive liver carcinogenesis (Emelyanov and Parinov, 2008; Nguyen et al., 2012). In this system, the transcriptional activator expressed by the transgenic driver is a ligand-dependent chimeric transcription factor, termed the LexPR transactivator. The LexPR transactivator is a fusion protein containing the DNA-binding domain (DBD) of the bacterial LexA repressor, a truncated ligand-binding domain (LBD) of the human progesterone receptor and the activation domain of NF-κB/p65 protein. The effector transgenic fish line harbours the operator-promoter sequence, consisting of the synthetic LexA operator fused to a minimal 35S promoter sequence from the Cauliflower mosaic virus, upstream of the reporter gene. In the presence of the progesterone agonist, mifepristone, which binds within the LexPR transactivator, LexPR activates the transcription of the target reporter gene by binding to the LexA-binding sites within the LexA operon (LexOP) positioned upstream (Fig. 1A).

In our system, we placed the LexPR transactivator, followed by Cerulean reporter, under the control of the melanocyte-specific promoters *kita* and *mitfa* using bacterial artificial chromosome (BAC)-mediated transgenesis (Fig. S1). Two melanocyte-specific driver lines [*TgBAC(kita:LexPR-Cerulean)^ox123^* (hereafter referred to as *kita:LexPR-Cerulean*) and *TgBAC(mitfa:LexPR-Cerulean)^ox124^* (hereafter referred to as *mitfa:LexPR-Cerulean*)] were generated (Fig. 1B,C).

To generate the effector lines, different Ras oncogenes (*NRas*^Q61K^, *HRas*^G12V^ and *KRas*^G12V^) fused to an mCherry fluorescent reporter were cloned downstream of the LexOP. The effector cassettes were placed within a non-autonomous maize dissociation (Ds) element, which allows for effective activator (Ac)-mediated genomic integration using the transposase Ac/Ds (AcDs) system (Emelyanov et al., 2006) (Fig. S1). This system is highly efficient and results in multiple independent integrations into the zebrafish genome, thus allowing for high-level expression of the Ras effectors activated by LexPR in the context of oncogenic transformation. Three Ras-effector lines were generated: *Tg(Crysβ:ECFP-LexOP:mCherry-NRas^*Q61K*^)^ox125^* (hereafter referred to as *LexOP:mCherry-NRas*^Q61K^), *Tg(Crysβ:ECFP-LexOP:mCherry-HRas^*G12V*^)^ox126^* and *Tg(Crysβ:ECFP-LexOP:mCherry-KRas^*G12V*^)^ox127^*. The Ras-effector lines all contain the selection marker enhanced cyan fluorescent protein (ECFP) under the control of the lens-specific zebrafish *crysβ* promoter (*crystallin betaβ*), which results in blue fluorescence in the lens. This can be used to screen for transgene integration, without driving the oncogene (Fig. 1D).

When a driver line is crossed to an effector line, in the presence of the synthetic progesterone ligand, mifepristone, mCherry–Ras-oncogene is transcribed only in melanocytes. Two different melanocyte-specific driver lines (*kita* and *mitfa*) and three different mCherry–Ras-oncogene lines were generated in this study, paving the way for multiple combinations of the system. We characterised two of these lines, *Tg(kita:LexPR-Cerulean;Crysβ:ECFP-LexOP:mCherry-NRas^*Q61K*^)^ox129^* (Fig. 1E) and *Tg(mitfa:LexPR-Cerulean; Crysβ:ECFP-LexOP:mCherry-NRas^*Q61K*^)^ox130^* (Fig. 1F) (hereafter referred to as *kita:LexPR;LexOP:NRas*^Q61K^ and *mitfa:LexPR;LexOP:NRas*^Q61K^, respectively) and showed that, when embryos positive for both alleles were reared in the presence of mifepristone, melanocyte-specific expression of the mCherry-NRas fusion protein was detected. Transformed melanocytes are seen in pairs or clumps of oncogene-activated red cells (as shown by the white arrowheads in Fig. 1G-G‴), indicative of their proliferative potential. In the *mitfa:mCherry-NRas*^Q61K^ genotype, low levels of melanin were occasionally observed in melanocytes expressing mCherry (data not shown).

**Fig. 2.**
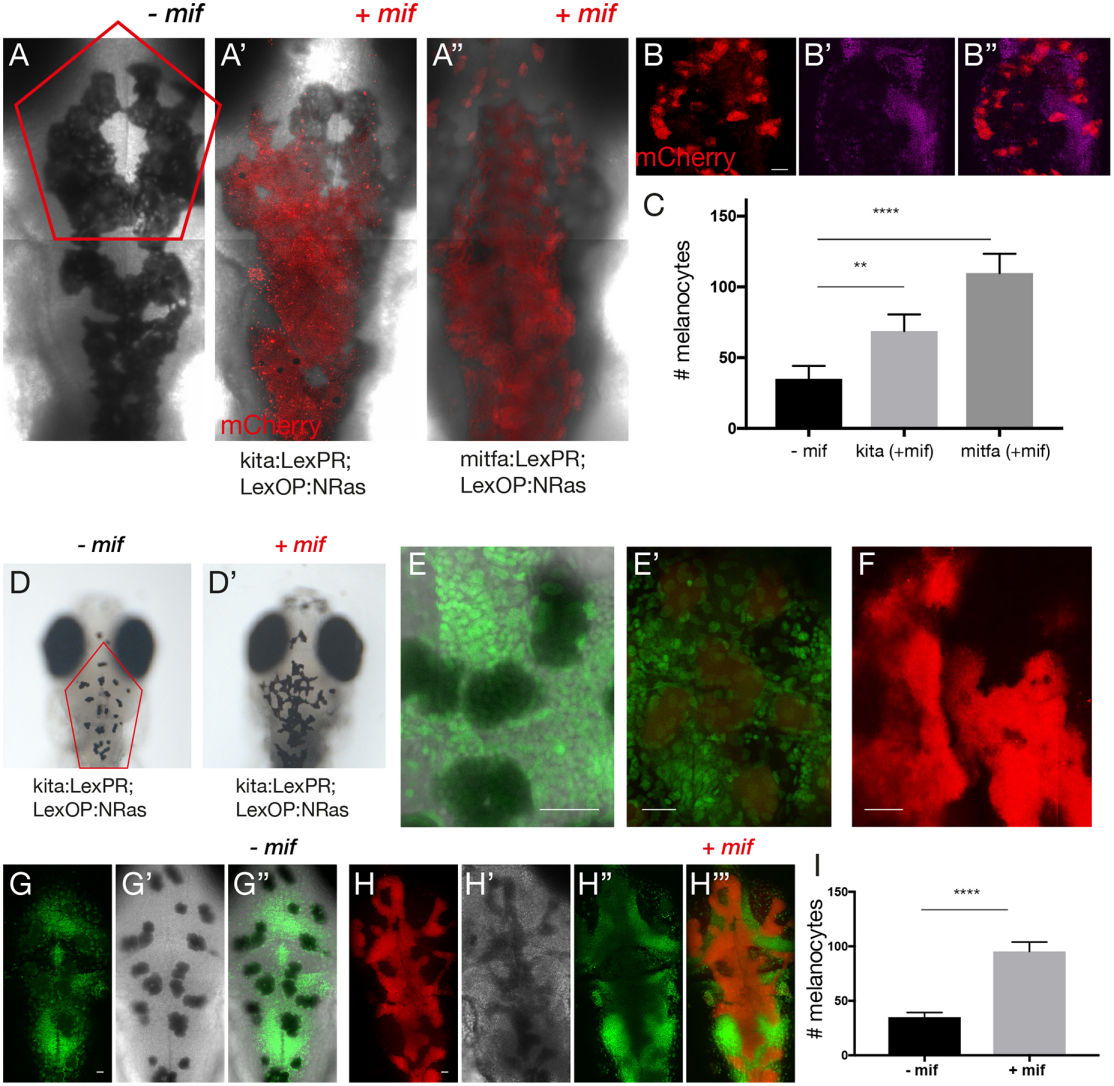
**Morphology and proliferative capacity of oncogene-activated melanocytes.** (A-A″) Composite images show confocal *z*-stack projection of the dorsal views of the head of 5-dpf larvae without (A) and with mifepristone-dependent *mCherry*-*NRas*^Q61K^ activation (red) in *Tg(kita:LexPR-Cerulean;LexOP:mCherry-NRas)^ox12^* (A′) and *Tg(mitfa:LexPR-Cerulean;LexOP:mCherry-NRas)^ox130^* (A″). The melanocyte-specific expression of *mCherry*-*NRas* in transgenic larvae harbouring both a transactivation driver and oncogene effector alleles was activated by addition of 1 μM mifepristone to the embryo rearing solution starting at 24 hpf. (B-B″) Representative image of the procedure used to facilitate counting of melanocytes shows a confocal *z*-stack projection of melanocytes (red) with a single plane of nuclear stain (magenta). (C) Quantification of the number of melanocytes in the region indicated by the red outline in A, comparing larvae from experimental (+mif) kita:LexPR;LexOP:NRas^Q61K^ (*n*=8) and mitfa:LexPR;LexOP:NRas^Q61K^ (*n*=8) to controls (−mif, *n*=7). Graph shows means±s.e.m. Statistical significance was determined by two-tailed unpaired Student's *t*-test with Welch's correction. (D,D′) Dorsal views of the head of 12-dpf larvae without (D) and with (D′) mifepristone-dependent *mCherry*-*NRas*^Q61K^ activation in *Tg(kita:LexPR-Cerulean;LexOP:mCherry-NRas)^ox12^*. (E,E′) Proliferative activity of activated melanocytes (red) (E′) shown with a nuclear stain (green) as compared to controls (E). (F) Confocal *z*-stack projection of melanocytes (red). (G,H) Representative image of the method used to facilitate counting of melanocytes shows a confocal *z*-stack projection of melanocytes (red) with a single plane of nuclear stain (green) in the absence (G) and presence (H) of mifepristone. (I) Quantification of the number of melanocytes in the region indicated by the red outline in D, comparing larvae from *kita:LexPR;LexOP:NRas*^Q61K^ (*n*=8) with and without oncogene activation (+/−mif). Graph shows means±s.e.m. Statistical significance was determined by two-tailed unpaired Student's *t*-test with Welch's correction. **P<0.01; ****P<0.0001. Scale bars: (B) 25 μm; (E) 31 μm; (E′) 27 μm; (F) 28 μm; (G) 25 μm and (H) 25 μm.

To assess and characterise the effect of *mitfa*- and *kita*-targeted oncogene activation, we compared the proliferative potential and morphology of control and activated melanocytes. We performed this analysis at two different time points: at 5 days post-fertilisation (dpf) (Fig. 2A-C), when the melanocytes are likely to be post-mitotic as the embryonic pigmentation pattern is thought to be largely complete at 48 h post-fertilisation (Kelsh et al., 2009, 1996) and at 12 dpf (Fig. 2D-I), just before the adult pigmentation patterning commences at 2 weeks post-fertilisation (Rawls et al., 2001).

To ensure optimal visualisation of the *mCherry-NRas*^Q61K^-expressing melanocytes at 5 dpf, embryos were partially bleached to ensure that excessive pigment in melanoblasts did not mask the mCherry signal and the signal amplified with an anti-Cherry antibody (Fig. 2A-B‴). A total of 5-9 *z*-stacks were acquired for the dorsal cranial region of each larvae and the melanocytes were counted within each corresponding plane (Fig. 2A-B‴). A nuclear stain (magenta) was used to aid in the visualisation of individual melanocytes (Fig. 2B). Strikingly, we observed that oncogene-activated melanocytes were present in clusters spanning multiple cell layers, in contrast to controls, where melanocytes lay within a single basal skin layer (Fig. 2A′-B″). We sought to quantify the degree of melanocyte proliferation in *kita:mCherry-NRas*^Q61K^ and *mitfa:mCherry-NRas*^Q61K^ embryos by precisely counting the total number of melanocytes within the outlined area in the presence and absence of mifepristone (+/− mif). Both *kita* and *mitfa* transgenic lines showed a statistically significant increase (*P*<0.01 and *P*<0.0001, respectively) in the number of melanocytes in the region of interest at 5 dpf (Fig. 2C). To ensure that the increase in melanocytes was a result of oncogene activation rather than an artefact of mifepristone application, we quantified the number of melanocytes in 5-dpf embryos reared as previously stated in the presence or absence of mifepristone at 5 dpf and could detect no difference (Fig. S2). To characterise the progression of the oncogene-activated cells at 12 dpf, we similarly examined both morphology and the proliferative state of the cranial melanocytes. As previously described, *kita:mCherry-NRas*^Q61K^ embryos were reared in the presence or absence of mifepristone. Whereas, in control embryos, the melanocytes appear to round up at 12 dpf, *mCherry-NRas*^Q61K^-expressing melanocytes exhibited an elongated fibroblast-like shape (Fig. 2D,D′, Fig. S3). Although larval melanophores are mostly replaced during larval-to-adult metamorphosis, some melanophores leave their initial position to form the stripes of the adult. Interestingly, these cells are characterised as small round black cells and are increasingly melanised (Parichy, 2006). To quantify the melanocytes, nuclear stain (green) was used to aid in the counting and visualisation of individual melanocytes cells (Fig. 2E,E′). Strikingly, cranial melanocytes were clearly present as multinucleated clusters compared to controls (Fig. 2E,E′). Melanocytes of control embryos were present in a single plane, in contrast to the oncogene-activated melanocytes, which were present in several overlapping layers of interlinked cells (Fig. 2F). Quantification of the melanocytes within the outlined area in Fig. 2D in the presence and absence of mifepristone at 12 dpf (+/− mif) (Fig. 2G-H‴) revealed a statistically significant increase (*P*<0.0001) in the number of melanocytes in the region of interest (Fig. 2I). All experiments are representative of two or three individual experiments.

### Establishment of a binary system for regulatory profiling in myeloid cells

**Fig. 3.**
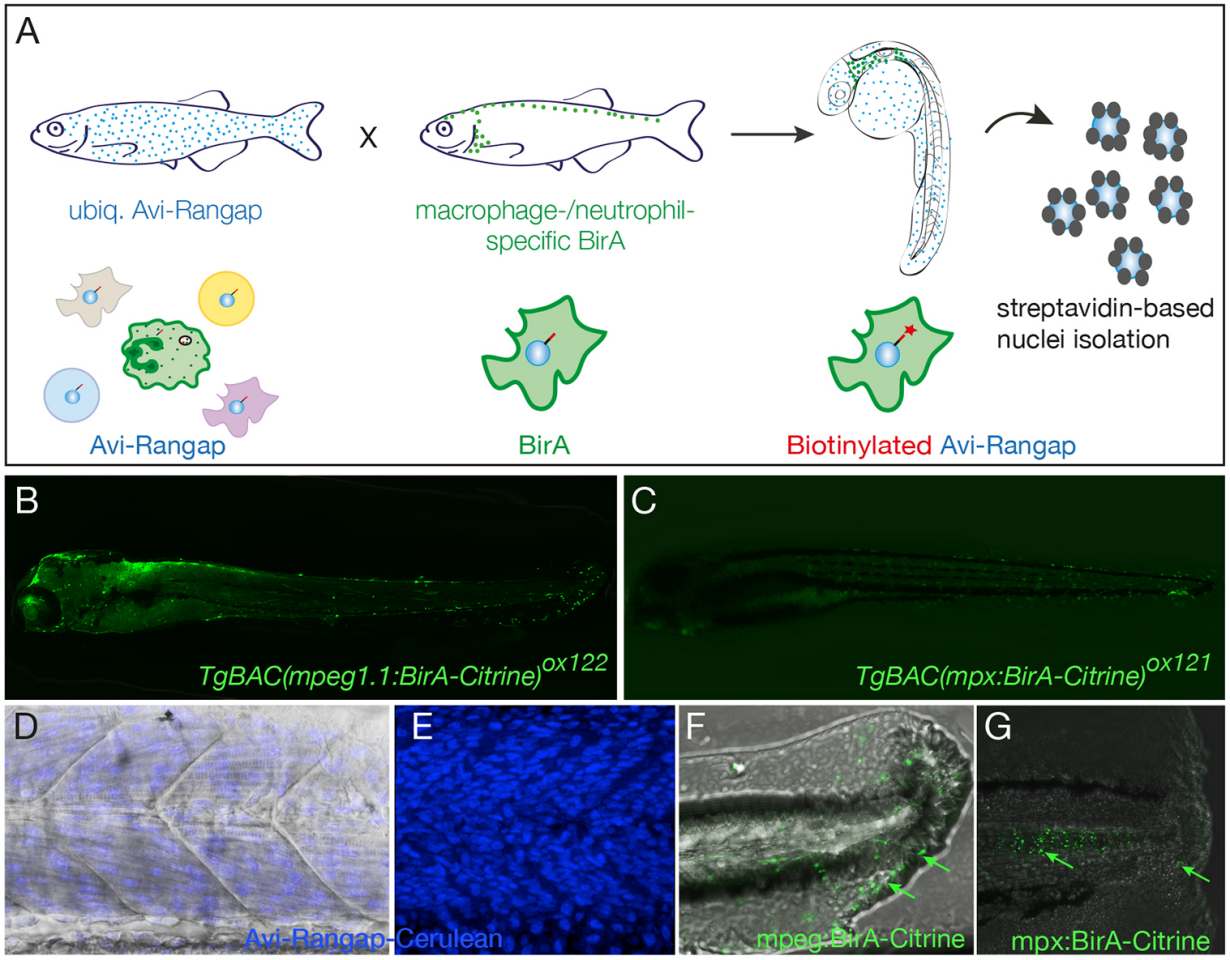
**Binary transgenic zebrafish model for regulatory profiling of myeloid cells.** (A) Schematic of myeloid nuclear biotagging system. When biotagging effector transgenic zebrafish line ubiquitously expressing Avi-tagged Rangap for biotinylation of nuclei is crossed to biotagging driver line expressing BirA in myeloid-specific manner, only the macrophage or neutrophil nuclei will be biotinylated. (B) Transgene expression in macrophage-specific BirA driver, *TgBAC(mpeg1:BirA-Citrine)^ox122^*, is amplified with anti-GFP–Alexa-Fluor-488 to show expression in macrophages. (C) *TgBAC(mpx:BirA-Citrine)^ox121^* biotagging transgenic driver shows neutrophil-specific expression. (D,E) Projection of confocal microscope images of *Tg(bactin:Avi-Cerulean-Rangap)^ct700a^* shows ubiquitous expression of the biotag effector specifically on nuclei, across somite region of the embryo, with (D) and without (E) bright field background. The images in D and E are taken in different embryos. (F,G) Tailfins of transgenic fish transected at 3 dpf show responding macrophages in *mpeg1:BirA-Citrine* (F) and neutrophils in *mpx:BirA-Citrine* (G) larvae at 5 hpi or 1 hpi, respectively, as indicated by green arrows.

Genome-wide analysis of the regulatory networks that govern the inflammatory response *in vivo* is complicated by the fact that relatively small numbers of responding cells are present within complex tissues. The current zebrafish lines for studying macrophage and neutrophil function make use of fluorescent reporters under the control of the *mpeg1* and *mpx* promoters, respectively (Ellett et al., 2011; Renshaw et al., 2006). These lines have largely been used for functional studies at the cellular level, allowing for a detailed examination of host-pathogen interactions, comparative analysis of macrophage and neutrophil behaviours responding to inflammation, and the dynamic interaction between the two cell types (Renshaw et al., 2006; Ellett et al., 2011). Based on the biotagging method (Trinh et al., 2017), we have developed a zebrafish model to study the gene regulatory landscape of macrophages and neutrophils by cell-specific *in vivo* biotagging of nuclei, thereby enabling us to obtain the active transcriptome of these cell types (Fig. 3A). In our system, the biotagging driver line harbours cell-specifically (macrophage or neutrophil) expressed *E. coli* biotin ligase BirA. Myeloid BirA driver lines *TgBAC(mpeg1:BirA-Citrine)^ox122^* [hereafter referred to as *mpeg1:BirA-Citrine* (Fig. 3B) and *TgBAC(mpx:BirA-Citrine)^ox121^* (hereafter referred to as *mpx:BirA-Citrine* (Fig. 3C) (described in Kenyon et al., 2017)] were generated using BAC-mediated transgenesis (Fig. S1). The previously characterised effector line *Tg(bactin:Avi-Cerulean-RanGap)^ct700a^* (hereafter referred to as *bactin:Avi-Cerulean-Rangap*) (Trinh et al., 2017) features ubiquitous expression of a zebrafish-compatible version of Avi-tagged nuclear-envelope-associated fusion protein. The Avi-tag is a 14-amino-acid minimal biotinylation substrate, specifically biotinylated by biotin holoenzyme synthetase, BirA, which adds a single biotin moiety onto a lysine residue within this peptide (de Boer et al., 2003). In addition to the biotinylatable Avi-tag, this effector protein consists of the carboxyl-terminal domain of Ran GTPase-activating protein1 (RanGap1), which acts as an outer-nuclear-envelope-targeting sequence, and a fluorescent variant Cerulean, allowing visualisation of the fusion protein (Fig. 3D,E). When the driver line is crossed to the effector line, in the specific cells (neutrophils or macrophages) that carry both biotagging alleles (driver and effector), the Avi-tagged fusion protein gets biotinylated and localises to the outer nuclear envelope. This process effectively results in the biotinylation of nuclei in a cell-specific (macrophage or neutrophil) fashion, allowing isolation of nuclei from these cell types using streptavidin-coated magnetic beads. Subsequently, the content of these nuclei can be analysed in a genome-wide fashion by next-generation sequencing (NGS) techniques. The use of *mpeg1:BirA-Citrine* and *mpx:BirA-Citrine* drivers combined with the *Avi-Cerulean-Rangap* effector offers the unique possibility to interrogate initial, often crucial, regulatory responses in specific cell types. Cell isolation protocols that are based on expression of fluorescent reporters always feature a delay between the time of expression of the cell-type-specific marker and the downstream analysis, because the fluorophore requires a certain amount of time to reach steady-state levels for visualisation or isolation by fluorescence-activated cell sorting (FACS). Given that cell-specific biotinylation begins very shortly after the cells start expressing *BirA*, the use of the biotagging system minimises such delays and enables faithful representation and readout of the relevant cell states. This is particularly crucial for analysis of cell populations in rapidly developing zebrafish embryos.

To show that the *mpeg1*/*mpx* driver lines generated in this study faithfully recapitulate larval inflammatory phenotypes, tailfin transections (Renshaw et al., 2006) were carried out at 3 dpf in *mpeg1:BirA-Citrine* and *mpx:BirA-Citrine* embryos, respectively. Injured specimens were fixed at 5 h post-injury (hpi) (*mpeg1:BirA-Citrine*) and 1 hpi (*mpx:BirA-Citrine*), and stained using anti-GFP antibody to amplify the Citrine signal and thus detect macrophages and neutrophils expressing *BirA* driver transgenes and their associated fluorescent reporter. Both *mpeg1:BirA-Citrine* (Fig. 3F) and *mpx:BirA-Citrine* (Fig. 3G) showed infiltration of macrophages and neutrophils to the site of injury.

### Generation of a double binary system to study the myeloid response to transformed melanocytes

**Fig. 4.**
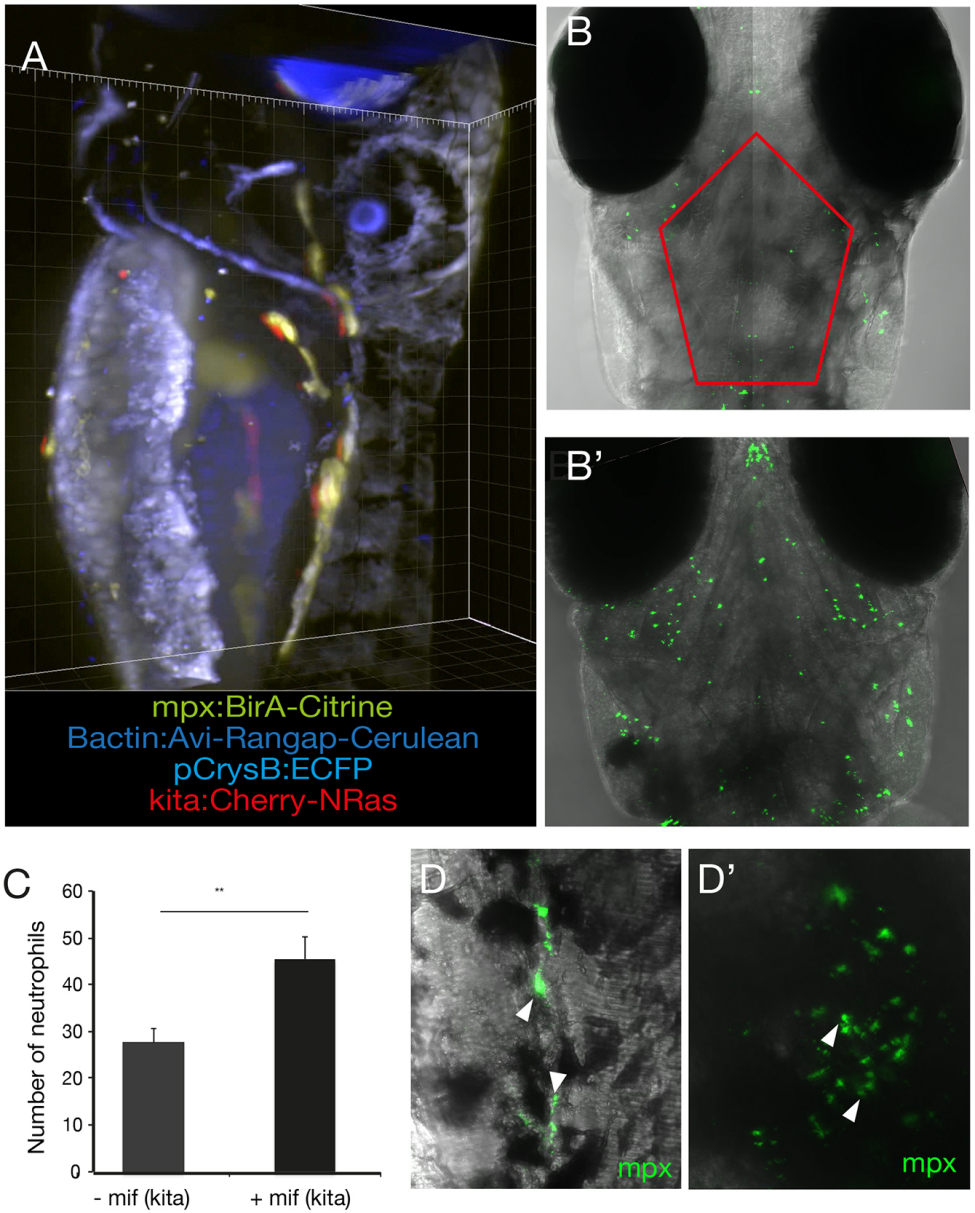
***mCherry*-*NRas*^K61Q^-activated melanocytes are immunogenic.** (A) Larvae at 5 dpi harbouring all four alleles (blue eye, blue nuclei, green neutrophils and red melanocytes) as captured on a Zeiss Z1 Light Sheet microscope. *kita:LexPR;LexOP:NRas*^Q61K^ were crossed to *mpx:BirA;bactin:Avi-Rangap*, and mCherry-NRas activated by addition of mifepristone. (B) The melanocyte-specific oncogene *NRas*^K61Q^ was activated by addition of mifepristone to the E3 medium in *Tg(kita:LexPR-Cerulean;pCrysβ:ECFP-LexOP:mCherry-NRas^*Q61K*^)^ox129^* embryos at 24 hpf. At 5 dpi, composite confocal images show neutrophils (green) as detected with an anti-mpx antibody in stage-matched controls (−mif) (B) and embryos where the oncogene had been activated (+mif) (B′). Images are a maximum intensity projection of 11 *z*-stack projections captured from the surface of the embryo to a depth of 100 μm. (C) Quantification of the number of neutrophils in the region of cranial melanocytes in the outlined area in B in control larvae (−mif, *n*=8) and following oncogene activation (+mif, *n*=8). Graph shows means±s.e.m. Statistical significance was determined by two-tailed unpaired Student's *t*-test with Welch's correction. **P<0.01. (D) High-resolution image of neutrophils in the tail (D) and head (D′) of 5-dpf zebrafish larvae following oncogene activation at 24 hpf, as shown by the arrowheads.

To study the initial myeloid response to oncogene-transformed melanocytes, we have generated a double binary system in zebrafish by coupling the inducible LexPR driver/effector to the biotagging system for nuclear transcriptional profiling of macrophages and neutrophils (Fig. 4A, Fig. S4). Since *NRas*^Q61K^ is the most relevant oncogene to human melanoma, with 30% of melanomas having contributing mutations in NRas (Ceol et al., 2008), it was prioritised for the purpose of this study. We hypothesised that, if *NRas*^Q61K^-transformed melanocytes generated by the inducible LexPR binary system were immunogenic, an increase in neutrophils in proximity to melanocytes following oncogene activation may be expected. *kita:LexPR;LexOP:NRas*^Q61K^ fish were in-crossed and embryos were grown to 24 hpf, at which point mifepristone is added to the embryo rearing medium in experimental embryos or omitted in non-treatment controls. Larvae were reared until 5 dpf, fixed and stained with an anti-mpx antibody to detect neutrophils. We chose to focus on the cranial region as this would be the focus of future experiments and capture only the neutrophils in the skin of the larvae in the vicinity of the melanocytes as compared to the controls. Images were captured as 11 *z*-stack projections to a depth of 100 μm from the surface of the embryo in *kita:LexPR;LexOP:NRas*^Q61K^ (+mif) and in genotype and stage-matched controls (−mif) larvae, and neutrophils were counted within the outlined area (Fig. 4B,B′). We observed a significant increase in the number of neutrophils when compared to stage- and genotype-matched controls with no oncogene activation (*P*<0.01) (Fig. 4C). Furthermore, high-resolution images show that neutrophils were detected in the vicinity as well as directly interacting with the melanocytes in the tail (Fig. 4D) and cranial region (Fig. 4D′) following oncogene activation.

### Transcriptional analysis of neutrophils in response to *NRas*^Q61K^-transformed melanocytes by *in vivo* biotagging

**Fig. 5.**
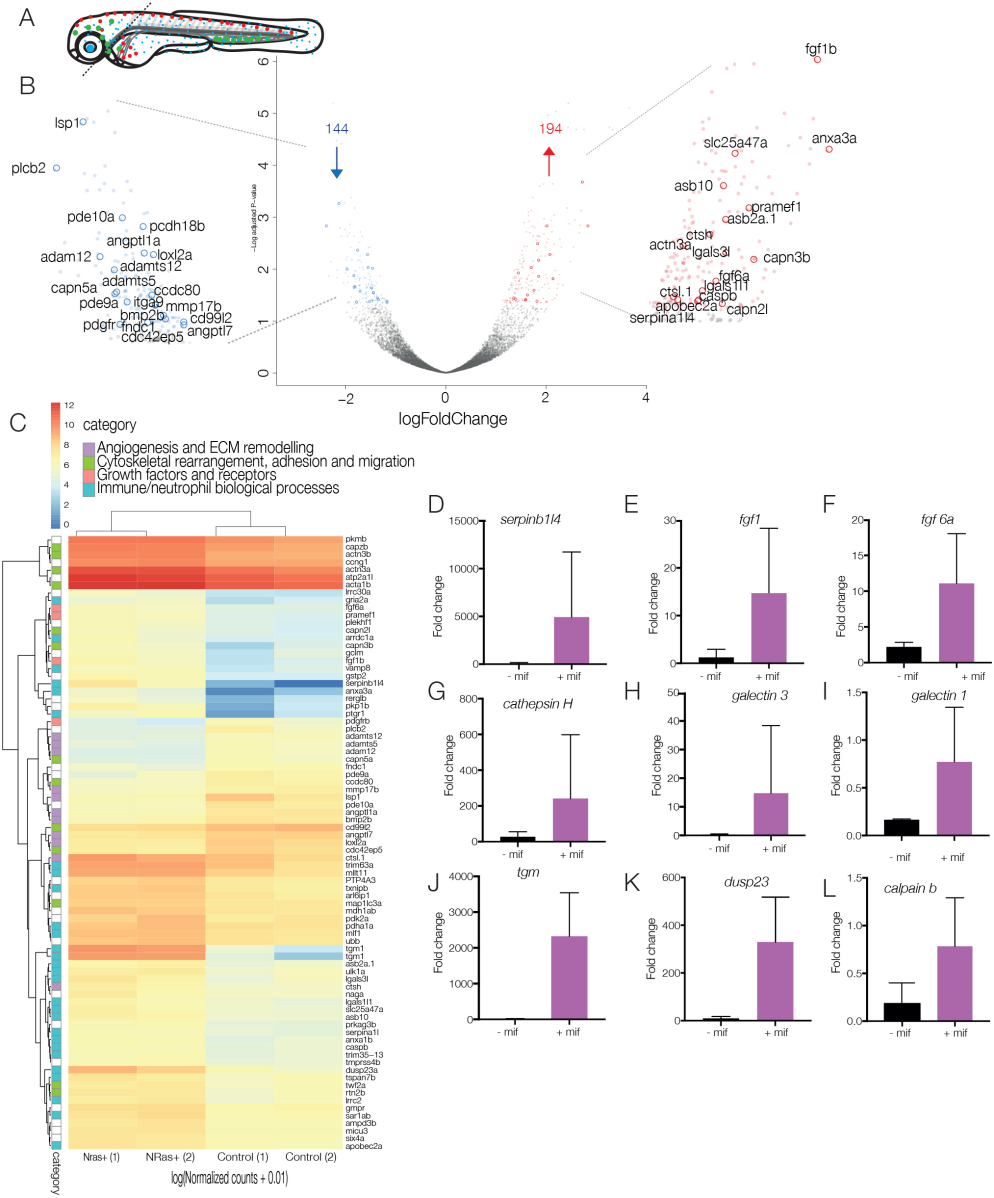
**Analysis of technical replicates to obtain differentially expressed genes.** (A) Schematic of dissection of 5-dpf larvae for nuclear profiling of neutrophils in the presence or absence of *NRas*^Q61K^-transformed cranial melanocytes. Cranial regions are dissected away from the trunk to eliminate neutrophils from the anterior yolk sac and ICM, enriching for responding neutrophils. (B) Volcano plot of differential expression analysis of activated *NRas*^Q61K^ and control embryos shows the relationship between *P-*value and log fold change (red, upregulated; blue, downregulated) shows 194 upregulated genes (red) and 144 downregulated genes (blue) in cranial neutrophils. (C) Heat map shows the log_10_ [normalised counts (NMCT) +0.01] of selected differentially expressed transcripts (adjusted *P-*value <0.05). Red, high expression; yellow, medium expression; blue, low expression. Selected differentially expressed transcripts were further classified into subcategories of ‘angiogenesis and ECM remodelling’ (purple), ‘cytoskeletal rearrangement, adhesion and migration’ (green), ‘growth factors and receptors’ (pink) and ‘immune/neutrophil biological processes’ (turquoise). (D-L) Bar graphs represent mRNA levels of upregulated transcripts in controls vs *NRas*^Q61K^-activated embryos as measured by qPCR in 2-3 replicate experiments. *serpinb1l4* (D), *fgf1* (E), *fgf6* (F), *cathepsin H* (G), *galectin 3* (H), *galectin 1* (I)*, tgm* (J), *dusp23* (K) and *calpain b* (L).

To test the efficacy of this model, we carried out a proof-of-concept study and performed transcriptional profiling of neutrophils responding to *NRas*^Q61K^-activated melanocytes, with the aim to identify components of the cellular response that might contribute to tumour progression. All subsequent experiments were carried out by crossing the *kita:LexPR;LexOP:NRas*^Q61K^ inducible melanoma system to the *mpx:BirA;bactin:Avi-Rangap* biotagging system, allowing isolation of neutrophil nuclei responding to oncogene activation. *NRas*^Q61K^ expression in the melanocytes of resulting embryos from this cross was driven by addition of mifepristone to the water at 24 hpf. Embryos expressing *mCherry-NRas*^Q61K^ in the melanocytes were selected for the profiling experiments, allowed to develop until 5 dpf and used for downstream analysis. Approximately 45 larvae from each condition – experimental (+mif) and control (−mif) – were used per experiment. A total of three biological replicates were collected. In each experiment, prior to nuclei isolation and analysis, cranial regions containing major foci of melanocyte oncogenic transformation were dissected to eliminate the anterior yolk sac and intermediate cell mass (ICM), thus enriching for responding neutrophils (Fig. 5A) (Bennett et al., 2001). By focussing our analysis on the isolated oncogene-activated mCherry-expressing regions in the head, we enriched the samples with environing neutrophils responding to transformed melanocytes. Moreover, *mCherry-NRas*^Q61K^-expressing goblet cells found in the trunk region were excluded from the analysis.

‘Biotagged’ nuclei were isolated from dissected tissue by nuclei pulldown. Nuclear RNA pools were extracted and libraries prepared using SMART-seq™ v4 technology for cDNA synthesis and amplification for small cell numbers followed by Illumina Nextera library preparation. Libraries were sequenced on an NGS platform.

Scatterplots of technical replicates show reproducibility between the three independent experiments (Fig. S5). Differential expression analysis comparing active transcription in neutrophil nuclei of embryos with *NRas*-transformed melanocytes versus control embryos identified 194 upregulated genes and 144 downregulated genes with a statistical significance cut off of *P*<0.05 (Fig. 5B,C, Fig. S3, Table S1).

The analysis of RNA sequencing (RNA-seq) datasets showed that a number of genes that may contribute to melanoma progression were upregulated in neutrophils upon engaging with transformed melanocytes. Those include mediators of tumour growth, angiogenesis and extracellular matrix (ECM) remodelling (Fig. 5D, Fig. S6), suggesting their potential pro-tumorigenic role in promoting progression to melanoma. In addition, we found significant enrichment for a number of factors involved in neutrophil migration, adhesion and cytoskeletal rearrangement as well as key factors involved in neutrophil biological processes (Fig. S6). To confirm the results of the RNA-seq analysis, we performed quantitative real-time PCR (qPCR) for a number of genes that were upregulated in the RNA-seq analysis. We detected an increase in *serpinb1l4* transcripts (Fig. 5D). SerpinB1 is an inhibitor of neutrophil serine proteases, including neutrophil granule proteins, elastase and cathepsin G (Benarafa et al., 2011). One potential mechanism by which neutrophils could eliminate transformed cells is by proteases stored in neutrophil granules. This data suggests that, instead, this mechanism is inhibited and neutrophils exhibit an immunosuppressive phenotype, allowing for melanocyte proliferation. We also detected an increase in members of the fibroblast growth factor (FGF) family, *fgf1* and *fgf6* (Fig. 5E,F), known to control cellular proliferation, survival, migration and differentiation. Uncontrolled FGF signalling, implicated in diverse tumour types, can drive tumorigenesis (Turner and Grose, 2010). Notably, we detect a statistically significant increase in cathepsin H (Fig. 5G). Cathepsins are a group of lysosomal proteinases or endopeptidases, of which different members can play a role in different tumorigenic processes, including proliferation, angiogenesis and metastasis (Tan et al., 2013). Furthermore, we see a statistically significant upregulation in members of the galectin family – *galectin 3* and *galectin 1* (Fig. 5H,I) – a group of proteins that bind β-galactoside and whose expression correlates with tumour development and invasiveness (Danguy et al., 2002). Other upregulated transcripts as confirmed by qPCR include *tgm*, *dusp23* and *calpain b* (Fig. 5J-L).

The prolonged exposure (21 days) to the progesterone agonist mifepristone has biological consequences at the level of gene expression in zebrafish larvae and adults (Bluthgen et al., 2013). To confirm that the transcriptional changes exhibited by the neutrophils were a direct result of oncogene-activated melanocytes rather than the 4-day exposure to mifepristone, nuclei pulldowns were carried out in mpx:BirA;bactin:Avi-Rangap fish. Embryos were grown to 24 hpf and mifepristone added to the E3 medium of the experimental batch, whereas control embryos were reared without mifepristone. Larvae were reared until 5 dpf, nuclear pulldowns performed and corresponding nuclear RNA pools extracted. Reverse transcription (RT)-qPCR assaying a number of candidate genes found to be upregulated in neutrophils upon the response to NRas activation in melanocytes was carried out. We found little or no change in transcript levels of *tgm1*, *fgf6a*, *fgf1*, *cathepsin L*, *gstp2*, *calpain b*, *galectin* *1*, *galectin* *3*, *dusp23*, *cathepsin H* and *serpinb14* upon the addition of mifepristone (Fig. S7A-K), suggesting that the results obtained after 4 days of exposure to mifepristone were a result of activation of *NRas*^Q61K^ rather than mifepristone exposure.

## DISCUSSION

Although progress has been made in understanding melanoma biology, the incidence of melanoma continues to increase and the prognosis for melanoma patients still remains poor (Anelli et al., 2009). Here, we report an inducible model to study the molecular mechanisms following oncogene activation in melanocytes that allows for the spatiotemporal control of oncogene expression in zebrafish melanocytes and thus the potential to study the earliest precursors of melanoma. Additionally, we have developed a binary model designed for regulatory nuclear profiling of myeloid cells. Coupled together, these systems provide the potential to understand the mechanisms by which macrophages and neutrophils may promote melanoma progression or conversely eliminate cancer cells as they establish themselves as the precursors of a tumour. Although not described in this study, the biotagging model can be extended to purification of ribosomes using the translating ribosomal affinity purification method (TRAP) based on the *in vivo* biotinylation of the Avi-tagged Rpl10 protein in zebrafish (Heiman et al., 2008; Trinh et al., 2017). A full understanding of the RNA landscape of neutrophils at the onset of melanoma would require both ribosomal and nuclear profiling to obtain the translated and active transcriptome, respectively (Trinh et al., 2017).

Importantly, in combination or as individual systems, both binary models presented in this study provide the potential to study different aspects of melanoma biology, including the transformation of melanocytes, myeloid immunity and immunotherapy. For example, the larval phenotype observed in this zebrafish melanoma model can provide a powerful platform for testing drug targets for preclinical testing of therapeutics. Because of the larval phenotype that we see, the mCherry-positive transformed cells can provide a direct biological readout in fast, easy-to-score chemical screens, aimed at finding compounds or drugs that may revert the overproliferation phenotype observed in melanocytes. Previous studies have shown that activated components of the Ras signalling pathway drive a tumour-promoting inflammatory response (Feng et al., 2010). However, these findings do not address the difference in the inflammatory response that different driver mutations may elicit. In our model, the same driver line can be crossed to multiple oncogene effector lines. This provides further opportunities to study mechanistic details of melanoma initiation and progression with different driver mutations.

In this proof-of-concept study, we chose to use this model system to profile the neutrophil response to *NRas*^Q61K^-oncogene-activated melanocytes using the *kita* driver. Neoplastic cells may only initiate tumour formation in the context of a supportive tumour microenvironment, largely influenced by myeloid cells. Although an immunosuppressive mechanism for neutrophils has been demonstrated in solid tumours, the role for neutrophils in tumour initiation largely remains to be explored. Although it is now accepted that tumour-associated neutrophils develop a pro-tumorigenic phenotype, largely driven by the presence of TGF-β, inhibition of TGF-β can modulate the cells to be tumouridical (Fridlender et al., 2009). In untreated tumours, neutrophils were reported to contribute to tumorigenesis by secreting factors that promote tumour growth, ECM remodelling and suppress the immune system (Galdiero et al., 2013). A study using a Tet-on inducible transgenic zebrafish line expressing oncogenic *KRas*^G12V^ in hepatocytes resulted in accelerated hepatocellular carcinoma (HCC) (Chew et al., 2014; Yan et al., 2017). The authors attributed the gender disparity of HCC, which occurs more frequently and aggressively in men than in women, to differences in cortisol levels, and reported a higher number of tumour-infiltrating neutrophils and macrophages in male zebrafish. After 7 days of doxycycline exposure in 3-month-old zebrafish, both infiltrating macrophages and neutrophils had a pro-tumour gene expression profile, although the effect was higher in male zebrafish than in their female counterparts (Yan et al., 2017).

We show here that neutrophils responding to early melanoma onset may provide an early source of Fgf1 and Fgf6. Given the multiple functional roles for FGF signalling in different tumours, the strong enrichment in FGF components in the neutrophils responding to oncogene-transformed melanocytes suggests a pro-tumorigenic role in promoting progression to melanoma. For example, uncontrolled FGF signalling in tumours can lead to both expansive tumour growth and progression to metastasis (Korc and Friesel, 2009). Furthermore, as FGF signalling can also promote angiogenesis, it is possible that the early role of FGFs in melanoma may be linked to angiogenesis, which not only promotes tumour growth but also the advancement from a pre-malignant to a malignant phenotype (Massi et al., 2010; Yang et al., 2015). In line with this interpretation, excessive angiogenesis, a hallmark of melanoma, and the progression from a radial growth phase to a vertical growth phase has been shown to require high angiogenic activity dependent on FGF1, FGF2 and VEGFA (Massi et al., 2010). In particular, FGF1 is a direct activator of phosphatidyl inositol 3-kinase (PI3K)-AKT signalling in endothelial cells known to initiate migration and invasion (Yang et al., 2015). These findings are particularly meaningful given that naturally targeting paracrine signalling in the form of growth and angiogenic factors is poised to provide exciting targets for cancer therapy.

Traditionally, cysteine cathepsin proteases are largely accepted as degradative enzymes of the lysosome. More recently, secreted cathepsins have emerged as potent effectors of multiple processes during tumour development, including the turnover and degradation of the ECM as well as processing or degradation of various growth factors, cytokines and chemokines. Consequently, cathepsins may promote tumour growth, tissue invasion and metastasis (Olson and Joyce, 2015). In a model of pancreatic islet carcinogenesis, deletion of cathepsin H significantly altered angiogenic switching of pre-malignant hyperplastic islets and ultimately a reduction in the number of tumours formed (Gocheva et al., 2010). Cathepsin H activity was largely attributed to macrophages in close proximity to the blood vessels (Gocheva et al., 2010). Here, we provide evidence that neutrophils may provide a source of cathepsin H in melanoma initiation.

Another interesting class of molecules implicated in tumour progression are the galectins, a family of lectin carbohydrate-binding proteins that function both intracellularly and extracellularly (Fortuna-Costa et al., 2014). Multiple examples exist in the literature for the role of galectins in tumour progression, including facilitating neoplastic transformation, tumour cell survival, angiogenesis and tumour metastasis (Liu and Rabinovich, 2005). Galectin 1, for example, increases cellular growth and exerts its effects by binding to the ECM as well as other cells (Liu and Rabinovich, 2005). In melanoma, galectin 1 has been thought to cause resistance of melanoma cells to cytotoxic stimuli and to enhance angiogenesis (Mathieu et al., 2012). Extracellular galectin 3 may influence tumour progression by impeding the endocytosis of key receptors, including TGFβ and EGF, while at the same time inducing endocytosis of β1 integrins (Furtak et al., 2001; Partridge et al., 2004). Extracellular galectin 3 secreted by tumour cells has also been shown to induce angiogenesis (Liu and Rabinovich, 2005). Furthermore, since galectins are expressed by immune cells, both intracellular and extracellular galectins may serve to regulate immune cell function (Liu and Rabinovich, 2005). Galectin 1 inhibits the release of inflammatory mediators by neutrophils and reduces transendothelial migration of the cells in response to inflammatory stimuli (Liu and Rabinovich, 2005; La et al., 2003). Therefore, targeting galectins derived from neutrophils in benign neoplasms may serve to prevent malignant transformation by promoting a pro-inflammatory environment. Galectin 3, on the other hand, can function as a chemokine attracting both monocytes and macrophages (Sano et al., 2000). In this capacity, galectin 3 may facilitate tumour progression driven by macrophages.

The zebrafish embryo has previously been used as a model organism to study the interactions between macrophages and neutrophils and oncogene-transformed melanocytes and goblet cells using a *kita:HRas*^G12V^ transgenic line (Feng et al., 2010). This study revealed a recruitment of both neutrophils and macrophages to oncogene-transformed cells. The authors reported frequent cytoplasmic tethers between the immune cells and transformed cells, including neutrophils, as well as phagocytosis of transformed cells by macrophages (Feng et al., 2010). In agreement with this data, we show, at the transcriptional level, an upregulation in signalling molecules involved in cell migration as well as cytoskeletal reorganisation in neutrophils.

In conclusion, we developed a double binary transgenic system for studying the myeloid response to the earliest precursors of melanoma and demonstrated it as a powerful resource, offering exciting potential to reveal inflammatory mediators that may contribute to tumour promotion and progression. This unbiased, genome-wide approach, carried out at the earliest stages of somatic cell transformation *in vivo*, is currently the only study of its kind. The ensuing gene set provides a list of interesting pathways and specific candidates to explore further in human melanoma biology. The results described here contribute to the growing body of evidence that suggests a tumour-promoting role of neutrophils in cancer biology and provides insight into the mechanisms by which neutrophils can be harnessed for immunotherapy.

## MATERIALS AND METHODS

### Zebrafish maintenance and strains

This study was carried out in compliance with local ethical approval from the University of Oxford (UK) and using procedures authorised by the UK Home Office in accordance with UK law (Animals Scientific Procedures Act 1986). Zebrafish were maintained as described previously (Westerfield, 2000). Wild-type embryos for transgenesis were obtained from AB or AB/TL mix strains.

### Transgenic fish line generation and maintenance

The pDs(cry:ECFP-LexOP:Cherry) construct was a kind gift from Dr Alexander Emelyanov and Dr Sergey Parinov (Emelyanov and Parinov, 2008). pCrysβ:ECFP-LexOP:mCherry-NRas^Q61K^, pCrysβ:ECFP-LexOP:mCherry-HRas^G12V^ and Crysβ:ECFP-LexOP:Cherry-KRas^G12V^ were cloned by In-Fusion cloning (Clontech, cat. no. 638909) using pBabe-NRas 61K (plasmid #12543; Addgene), mEGFP-HRas^G12V^ (plasmid #18666; Addgene) and pEFm.6 HA-KRas^v12^ (a gift from Prof. Xin Lu, Ludwig Institute for Cancer Research Oxford, Oxford, UK) were used as donor vectors. The optimised open reading frame (ORF) of the LexPR transactivator, which eliminates all putative donor and acceptor sites that could interfere with proper production of the *LexPR* transcript, was synthesised based on the coding sequence obtained from Dr Parinov (Temasek Life Sciences Laboratory, National Institute of Singapore, Singapore) and assembled using G-Blocks (Integrated DNA Technologies, BVBA, Leuven, Belgium). pGEM LexPR-2A-Cerulean-SV40pA-FRT-Kan-FRT donor plasmid for BAC transgenesis was cloned by In-Fusion cloning. pGEM BirA-2A-Citrine-SV40pA-FRT-Kan-FRT donor plasmid for BAC transgenesis was cloned by fusion PCR of HA-BirA-2A amplified from PMT-HA-BirA-2A-mCherryRas (Trinh et al., 2017) and inserted into pGEM-GFP-SV40pA-FRT-Kan-FRT where the GFP ORF was been replaced with Citrine. All plasmids generated for the purposes of this study are listed in Table S2 and available from Addgene (https://www.addgene.org/Tatjana_Sauka-Spengler/).

The *TgBAC(mpeg1:BirA-Citrine)^ox122^* and *TgBAC(kita/mitfa:LexPR-Cerulean)^ox123/ox124^* transgenic lines were generated by BAC-mediated transgenesis as previously described (Trinh et al., 2017). Briefly, PCR-amplified BirA-2A-Citrine-SV40pA-FRT-Kan-FRT and LexPR-2A-Cerulean-SV40pA-FRT-Kan-FRT cassettes were recombined into the first coding exon of a driver gene within the corresponding BAC clone (macrophage-specific *mpeg1* gene, and melanocyte-specific *mitfa* or *kita*, respectively). In a second recombination step, an iTol2-Ampicillin cassette (provided by Prof. Kawakami, National Institute of Genetics, Mishima, Japan) was introduced into the BAC backbone as previously published (Suster et al., 2011; Bussmann and Schulte-Merker, 2011). Wild-type embryos were injected at the one-cell stage with 200 ng/μl of purified BAC DNA and 100 ng/μl *tol2* transposase mRNA. Putative founders were outcrossed to wild-type fish and offspring screened for Citrine or Cerulean expression, in combination with PCR amplification of the transgene when expression levels were low. The full list of zebrafish transgenic lines generated and used in this study can be found in Table S3.

In transgenic embryos harbouring both a transactivation driver and oncogene effector allele, melanocyte-specific expression of mCherry–Ras-oncogene was activated by addition of 1 μM mifepristone (Sigma, cat. no. M8046) to the E3 medium and changed daily.

### Whole-mount immunofluorescence and imaging

Embryos/larvae were fixed in 4% PFA in phosphate buffered saline (PBS) (0.1 M, pH 7.4) for 1 h at room temperature (RT). Embryos were bleached (3% H_2_O_2_, 0.8% KOH and 0.5% Tween20) and post-fixed in 4% PFA in PBS for 1 h at RT. Embryos were then rinsed 3×15 min in PBT (2% DMSO, 0.5% Triton in PBS) and incubated in block solution (10% donkey serum in PBT) for 2 h at RT, followed by incubation with primary antibody (1:300 in block solution) overnight (O/N) at 4°C. Embryos/larvae were then washed 3-5 times for 1 h at RT, followed by an O/N wash at 4°C in PBT and then incubated with secondary antibody (1:1000 in PBT). Embryos were then washed 6-8 times for 1 h at RT+O/N at 4°C in PBT. The primary antibody used to detect neutrophils was rabbit anti-mpx (GeneTex, cat. no. GTX128379) and the secondary antibody used was Alexa-Fluor-488-conjugated anti-rabbit IgG (Life Technologies, cat. no. R37116). For amplification of mCherry signal we used Living Colors^®^ mCherry Monoclonal Antibody (1:200) (Clontech, cat. no. 632543) as primary and Alexa-Fluor-568-conjugated goat anti-mouse IgG (H+L) (Life Technologies, cat. no. A-11004) as secondary antibody. For quantification of melanocytes, the far-red nuclear counterstain RedDot1 (Cambridge Biosciences, BT40060) was used. Images of stained embryos/larvae were taken on an inverted Zeiss 780 confocal microscope or captured using the Zeiss Z1 Light Sheet microscope.

### Tail transection

At 3 dpf, larvae were anaesthetised with 0.02% 3-amino benzoic acidethylester (Tricaine) in E3 medium and tail transection was performed with a sterile scalpel.

### ‘Biotagged’ nuclei isolation

For nuclei isolation, zebrafish embryos were anaesthetised with 0.01% Tricaine and cranial regions dissected away from anterior yolk sac and ICM. Embryos were washed in hypotonic buffer H [20 mM HEPES (pH 7.9), 15 mM MgCl_2_, 10 mM KCl, 1 mM DTT and 1× complete protease inhibitor (Roche)] and subsequently re-suspended in 500 μl of buffer H. From here, nuclei isolation was carried out as previously described (Deal and Henikoff, 2010; Trinh et al., 2017). Following isolation, nuclei-beads were re-suspended in RNA lysis buffer from RNAqueous^®^-Micro Kit (Life Technologies, cat. no. AM1931).

### RNA extraction, library preparation and RNA-seq analysis

Total nuclear RNA was extracted using RNAqueous^®^-Micro Kit according to the manufacturer's instructions. RNA integrity was checked with an RNA Pico chip (Agilent Technologies, cat. no. 5067-1513) using the Agilent 2100 Bioanalyzer.

cDNA was synthesised and amplified from 100 pg to 1 ng of input RNA using SMART-seq™ v4 ultra-low input kit for RNA (Clontech Laboratories, cat. nos 634888, 634889, 634890, 634891, 634892, 634893 and 634894). Sequencing libraries were prepared using the Nextera XT DNA library preparation kit and NGS was performed on a NextSeq500 platform using a NextSeq™500 150 cycle High Output Kit (Illumina, cat. no. FC-404-1002) to generate 80-basepair paired end reads.

### RNA-seq analysis

Read quality was evaluated using FastQC (Andrews, 2011). Reads were mapped to the ‘Jul. 2014 Zv10/danRer10 assembly’ version of the zebrafish genome using STAR (v.2.4.2a) splice-aware aligner (Dobin et al., 2013). Count tables were generated using subread FeatureCounts (v1.4.5-p1q), with standard parameters (Liao et al., 2014). Differential expression was carried out using DESeq2 R package (Love et al., 2014). Data generated in this study were submitted to GEO (GSE96534).

### Quantitative PCR

RNA extraction and cDNA synthesis were carried out using the RNAqueous^®^-Micro Kit (Life Technologies, cat. no. AM1931) and Superscript III Reverse Transcriptase (Invitrogen, cat. no. 18080093), respectively. Quantitative PCR was performed using Fast SYBR Green Master Mix (Applied Biosystems, cat. no. 4385612) on a 7500 Fast Real-Time PCR system (Applied Biosystems). Gene-specific primers were used for tgm (5′-TGCGATTATGACTGCAAGCA-3′ and 3′-TCCAGCACTTCATCAGGTCA), fgf6a (5′-GCCAGACGGAAGGATAAACG-3′ and 3′-GACAAACAGCCCGCTTTTCA-5′), fgf1 (5′-AAAGCAGTGAAAGCAGGAGT-3′ and 3′-AGAAACACTCTTCATTCAGCACA-5′), cathepsin l (5′-CTGCTGCTTTTCTGGCTGTT-3′ and 3′-ACGGTGTGACTCTTCCTCTG-5′), gstp2 (5′-TCTCTTTGGACAGCTGCCTA-3′ and 3′-CGTTCTTTCCATACGCACCA-5′), calpain b (5′-AGCTCAGGACTCCTCTCTCT-3′ and 3′-CCTTGGCAGATGTCAGTCCT-5′), galectin 3 (5′-GACAGCCTGGACAACTGACT-3′ and 3′-AGAGGGAAATCAAGTGGCAC-5′), galectin 1 (5′-TGTGCTTATACAGAATATGGCCT-3′ and 3′-GCGATGTCTTCAGAGCTGTT-5′), serpinb1l4 (5′-CTCGGTGCCAAAGGAAACAC-3′ and 3′-CCATTTGGGCTTCTGGGTTG-5′), cathepsin H (5′-CGTTGTACACCGAGGAAGATG-3′ and 3′-GGTCAATCCTCTTCTTGTTCTCC-5′) and dusp23 (5′-CGAGTCGCAGTCAAATCCTG-3′ and 3′-CGTCCTGCCGTGACCATG-5′). Expression levels were normalised to β-actin.

### Statistical analysis

Graphs and statistical analyses were generated by GraphPad prism, version 6. For quantification of the melanocytes and neutrophils, statistical significance was determined by two-tailed unpaired Student's *t*-test with Welch's correction. The following *P*-values are indicated in the figures: not significant, *P>*0.05; **P<0.01; ****P<0.0001.

## Supplementary Material

Supplementary information

## References

[DMM030056C1] Akitake C. M., Macurak M., Halpern M. E. and Goll M. G. (2011). Transgenerational analysis of transcriptional silencing in zebrafish. 352, 191-201. 10.1016/j.ydbio.2011.01.002 PMC3065955 21223961

[DMM030056C2] Andrews S. (2011). *FastQC—a quality control tool for high throughput sequence data* [Online] Babraham Bioinformatics. Available: http://www.bioinformatics.babraham.ac.uk/projects/fastqc/.

[DMM030056C3] Anelli V., Santoriello C., Distel M., Köster R. W., Ciccarelli F. D. and Mione M. (2009). Global repression of cancer gene expression in a zebrafish model of melanoma is linked to epigenetic regulation. 6, 417-424. 10.1089/zeb.2009.0612 20047469

[DMM030056C4] Benarafa C., Lecuyer T. E., Baumann M., Stolley J. M., Cremona T. P. and Remold-O'donnell E. (2011). SerpinB1 protects the mature neutrophil reserve in the bone marrow. 90, 21-29. 10.1189/jlb.0810461 PMC3114599 21248149

[DMM030056C5] Bennett C. M., Kanki J. P., Rhodes J., Liu T. X., Paw B. H., Kieran M. W., Langenau D. M., Delahaye-Brown A., Zon L. I., Fleming M. D. et al. (2001). Myelopoiesis in the zebrafish, Danio rerio. 98, 643-651. 10.1182/blood.V98.3.643 11468162

[DMM030056C6] Blair G. E. and Cook G. P. (2008). Cancer and the immune system: an overview. 27, 5868 10.1038/onc.2008.277 18836467

[DMM030056C7] Bluthgen N., Sumpter J. P., Odermatt A. and Fent K. (2013). Effects of low concentrations of the antiprogestin mifepristone (RU486) in adults and embryos of zebrafish (Danio rerio): 2. Gene expression analysis and in vitro activity. 144-145, 96-104. 10.1016/j.aquatox.2013.09.030 24177212

[DMM030056C8] Bussmann J. and Schulte-Merker S. (2011). Rapid BAC selection for tol2-mediated transgenesis in zebrafish. 138, 4327-4332. 10.1242/dev.068080 21865323

[DMM030056C9] Ceol C. J., Houvras Y., White R. M. and Zon L. I. (2008). Melanoma biology and the promise of zebrafish. 5, 247-255. 10.1089/zeb.2008.0544 PMC2784934 19133823

[DMM030056C10] Chew T. W., Liu X. J., Liu L., Spitsbergen J. M., Gong Z. and Low B. C. (2014). Crosstalk of Ras and Rho: activation of RhoA abates Kras-induced liver tumorigenesis in transgenic zebrafish models. 33, 2717-2727. 10.1038/onc.2013.240 23812423

[DMM030056C11] Danguy A., Camby I. and Kiss R. (2002). Galectins and cancer. 1572, 285-293. 10.1016/S0304-4165(02)00315-X 12223276

[DMM030056C12] de Boer E., Rodriguez P., Bonte E., Krijgsveld J., Katsantoni E., Heck A., Grosveld F. and Strouboulis J. (2003). Efficient biotinylation and single-step purification of tagged transcription factors in mammalian cells and transgenic mice. 100, 7480-7485. 10.1073/pnas.1332608100 PMC164612 12802011

[DMM030056C13] de Visser K. E., Eichten A. and Coussens L. M. (2006). Paradoxical roles of the immune system during cancer development. 6, 24-37. 10.1038/nrc1782 16397525

[DMM030056C14] Deal R. B. and Henikoff S. (2010). A simple method for gene expression and chromatin profiling of individual cell types within a tissue. 18, 1030-1040. 10.1016/j.devcel.2010.05.013 PMC2905389 20627084

[DMM030056C15] Dobin A., Davis C. A., Schlesinger F., Drenkow J., Zaleski C., Jha S., Batut P., Chaisson M. and Gingeras T. R. (2013). STAR: ultrafast universal RNA-seq aligner. 29, 15-21. 10.1093/bioinformatics/bts635 PMC3530905 23104886

[DMM030056C16] Dovey M., White R. M. and Zon L. I. (2009). Oncogenic NRAS cooperates with p53 loss to generate melanoma in zebrafish. 6, 397-404. 10.1089/zeb.2009.0606 PMC2943216 19954345

[DMM030056C17] Ellett F., Pase L., Hayman J. W., Andrianopoulos A. and Lieschke G. J. (2011). mpeg1 promoter transgenes direct macrophage-lineage expression in zebrafish. 117, e49-e56. 10.1182/blood-2010-10-314120 PMC3056479 21084707

[DMM030056C18] Emelyanov A. and Parinov S. (2008). Mifepristone-inducible LexPR system to drive and control gene expression in transgenic zebrafish. 320, 113-121. 10.1016/j.ydbio.2008.04.042 18544450

[DMM030056C19] Emelyanov A., Gao Y., Naqvi N. I. and Parinov S. (2006). Trans-kingdom transposition of the maize dissociation element. 174, 1095-1104. 10.1534/genetics.106.061184 PMC1667081 16951067

[DMM030056C20] Engblom C., Pfirschke C. and Pittet M. J. (2016). The role of myeloid cells in cancer therapies. 16, 447-462. 10.1038/nrc.2016.54 27339708

[DMM030056C21] Feng Y., Santoriello C., Mione M., Hurlstone A. and Martin P. (2010). Live imaging of innate immune cell sensing of transformed cells in zebrafish larvae: parallels between tumor initiation and wound inflammation. 8, e1000562 10.1371/journal.pbio.1000562 PMC3001901 21179501

[DMM030056C22] Feng Y., Renshaw S. and Martin P. (2012). Live imaging of tumor initiation in zebrafish larvae reveals a trophic role for leukocyte-derived PGE(2). 22, 1253-1259. 10.1016/j.cub.2012.05.010 PMC3398414 22658594

[DMM030056C23] Fortuna-Costa A., Gomes A. M., Kozlowski E. O., Stelling M. P. and Pavao M. S. (2014). Extracellular galectin-3 in tumor progression and metastasis. 4, 138 10.3389/fonc.2014.00138 PMC4058817 24982845

[DMM030056C24] Fridlender Z. G., Sun J., Kim S., Kapoor V., Cheng G., Ling L., Worthen G. S. and Albelda S. M. (2009). Polarization of tumor-associated neutrophil phenotype by TGF-beta: “N1” versus “N2” TAN. 16, 183-194. 10.1016/j.ccr.2009.06.017 PMC2754404 19732719

[DMM030056C25] Furtak V., Hatcher F. and Ochieng J. (2001). Galectin-3 mediates the endocytosis of beta-1 integrins by breast carcinoma cells. 289, 845-850. 10.1006/bbrc.2001.6064 11735123

[DMM030056C26] Galdiero M. R., Bonavita E., Barajon I., Garlanda C., Mantovani A. and Jaillon S. (2013). Tumor associated macrophages and neutrophils in cancer. 218, 1402-1410. 10.1016/j.imbio.2013.06.003 23891329

[DMM030056C27] Gocheva V., Chen X., Peters C., Reinheckel T. and Joyce J. A. (2010). Deletion of cathepsin H perturbs angiogenic switching, vascularization and growth of tumors in a mouse model of pancreatic islet cell cancer. 391, 937-945. 10.1515/bc.2010.080 PMC3126667 20731543

[DMM030056C28] Goll M. G., Anderson R., Stainier D. Y. R., Spradling A. C. and Halpern M. E. (2009). Transcriptional silencing and reactivation in transgenic zebrafish. 182, 747-755. 10.1534/genetics.109.102079 PMC2710156 19433629

[DMM030056C29] Heiman M., Schaefer A., Gong S., Peterson J. D., Day M., Ramsey K. E., Suárez-Fariñas M., Schwarz C., Stephan D. A., Surmeier D. J. et al. (2008). A translational profiling approach for the molecular characterization of CNS cell types. 135, 738-748. 10.1016/j.cell.2008.10.028 PMC2696821 19013281

[DMM030056C30] Kelsh R. N., Brand M., Jiang Y. J., Heisenberg C. P., Lin S., Haffter P., Odenthal J., Mullins M. C., van Eeden F. J., Furutani-Seiki M. et al. (1996). Zebrafish pigmentation mutations and the processes of neural crest development. 123, 369-389. 10.1242/dev.123.1.369 9007256

[DMM030056C31] Kelsh R. N., Harris M. L., Colanesi S. and Erickson C. A. (2009). Stripes and belly-spots -- a review of pigment cell morphogenesis in vertebrates. 20, 90-104. 10.1016/j.semcdb.2008.10.001 PMC2744437 18977309

[DMM030056C32] Kenyon A., Gavriouchkina D., Zorman J., Napolitani G., Cerundolo V. and Sauka-Spengler T. (2017). Active nuclear transcriptome analysis reveals inflammasome-dependent mechanism for early neutrophil response to Mycobacterium marinum. 7, 6505 10.1038/s41598-017-06099-x PMC5529371 28747644

[DMM030056C33] Korc M. and Friesel R. E. (2009). The role of fibroblast growth factors in tumor growth. 9, 639-651. 10.2174/156800909789057006 PMC3664927 19508171

[DMM030056C34] La M., Cao T. V., Cerchiaro G., Chilton K., Hirabayashi J., Kasai K., Oliani S. M., Chernajovsky Y. and Perretti M. (2003). A novel biological activity for galectin-1: inhibition of leukocyte-endothelial cell interactions in experimental inflammation. 163, 1505-1515. 10.1016/S0002-9440(10)63507-9 PMC1868297 14507657

[DMM030056C35] Liao Y., Smyth G. K. and Shi W. (2014). featureCounts: an efficient general purpose program for assigning sequence reads to genomic features. 30, 923-930. 10.1093/bioinformatics/btt656 24227677

[DMM030056C36] Liu F.-T. and Rabinovich G. A. (2005). Galectins as modulators of tumour progression. 5, 29-41. 10.1038/nrc1527 15630413

[DMM030056C37] Love M. I., Huber W. and Anders S. (2014). Moderated estimation of fold change and dispersion for RNA-seq data with DESeq2. 15, 550 10.1186/s13059-014-0550-8 PMC4302049 25516281

[DMM030056C38] Massi D., Landriscina M., Piscazzi A., Cosci E., Kirov A., Paglierani M., di Serio C., Mourmouras V., Fumagalli S., Biagioli M. et al. (2010). S100A13 is a new angiogenic marker in human melanoma. 23, 804-813. 10.1038/modpathol.2010.54 PMC2882157 20208480

[DMM030056C39] Mathieu V., de Lassalle E. M., Toelen J., Mohr T., Bellahcène A., van Goietsenoven G., Verschuere T., Bouzin C., Debyser Z., de Vleeschouwer S. et al. (2012). Galectin-1 in melanoma biology and related neo-angiogenesis processes. 132, 2245-2254. 10.1038/jid.2012.142 22622427

[DMM030056C40] Michailidou C., Jones M., Walker P., Kamarashev J., Kelly A. and Hurlstone A. F. L. (2009). Dissecting the roles of Raf- and PI3K-signalling pathways in melanoma formation and progression in a zebrafish model. 2, 399-411. 10.1242/dmm.001149 19470611

[DMM030056C41] Nguyen A. T., Emelyanov A., Koh C. H. V., Spitsbergen J. M., Parinov S. and Gong Z. (2012). An inducible kras(V12) transgenic zebrafish model for liver tumorigenesis and chemical drug screening. 5, 63-72. 10.1242/dmm.008367 PMC3255544 21903676

[DMM030056C42] Olson O. C. and Joyce J. A. (2015). Cysteine cathepsin proteases: regulators of cancer progression and therapeutic response. 15, 712-729. 10.1038/nrc4027 26597527

[DMM030056C43] Parichy D. M. (2006). Evolution of danio pigment pattern development. 97, 200-210. 10.1038/sj.hdy.6800867 16835593

[DMM030056C44] Partridge E. A., le Roy C., di Guglielmo G. M., Pawling J., Cheung P., Granovsky M., Nabi I. R., Wrana J. L. and Dennis J. W. (2004). Regulation of cytokine receptors by Golgi N-glycan processing and endocytosis. 306, 120-124. 10.1126/science.1102109 15459394

[DMM030056C45] Patton E. E., Widlund H. R., Kutok J. L., Kopani K. R., Amatruda J. F., Murphey R. D., Berghmans S., Mayhall E. A., Traver D., Fletcher C. D. M. et al. (2005). BRAF mutations are sufficient to promote nevi formation and cooperate with p53 in the genesis of melanoma. 15, 249-254. 10.1016/j.cub.2005.01.031 15694309

[DMM030056C46] Rawls J. F., Mellgren E. M. and Johnson S. L. (2001). How the zebrafish gets its stripes. 240, 301-314. 10.1006/dbio.2001.0418 11784065

[DMM030056C47] Renshaw S. A., Loynes C. A., Trushell D. M. I., Elworthy S., Ingham P. W. and Whyte M. K. B. (2006). A transgenic zebrafish model of neutrophilic inflammation. 108, 3976-3978. 10.1182/blood-2006-05-024075 16926288

[DMM030056C48] Sano H., Hsu D. K., Yu L., Apgar J. R., Kuwabara I., Yamanaka T., Hirashima M. and Liu F.-T. (2000). Human galectin-3 is a novel chemoattractant for monocytes and macrophages. 165, 2156-2164. 10.4049/jimmunol.165.4.2156 10925302

[DMM030056C49] Santoriello C., Gennaro E., Anelli V., Distel M., Kelly A., Köster R. W., Hurlstone A. and Mione M. (2010). Kita driven expression of oncogenic HRAS leads to early onset and highly penetrant melanoma in zebrafish. 5, e15170 10.1371/journal.pone.0015170 PMC3000817 21170325

[DMM030056C50] Stern H. M. and Zon L. I. (2003). Cancer genetics and drug discovery in the zebrafish. 3, 533-539. 10.1038/nrc1126 12835673

[DMM030056C51] Suster M. L., Abe G., Schouw A. and Kawakami K. (2011). Transposon-mediated BAC transgenesis in zebrafish. 6, 1998-2021. 10.1038/nprot.2011.416 22134125

[DMM030056C52] Tan G.-J., Peng Z.-K., Lu J.-P. and Tang F.-Q. (2013). Cathepsins mediate tumor metastasis. 4, 91-101. 10.4331/wjbc.v4.i4.91 PMC3856311 24340132

[DMM030056C53] Trinh L. A., Chong-Morrison V., Gavriouchkina D., Hochgreb-Hägele T., Senanayake U., Fraser S. E. and Sauka-Spengler T. (2017). Biotagging of specific cell populations in Zebrafish reveals gene regulatory logic encoded in the nuclear transcriptome. 19, 425-440. 10.1016/j.celrep.2017.03.045 PMC5400779 28402863

[DMM030056C54] Turner N. and Grose R. (2010). Fibroblast growth factor signalling: from development to cancer. 10, 116-129. 10.1038/nrc2780 20094046

[DMM030056C55] Vallone D., Santoriello C., Gondi S. B. and Foulkes N. S. (2007). Basic protocols for zebrafish cell lines: maintenance and transfection. 362, 429-441. 10.1007/978-1-59745-257-1_35 17417032

[DMM030056C56] Westerfield M. (2000). . Eugene, OR, USA: University of Oregon Press.

[DMM030056C57] White R., Rose K. and Zon L. (2013). Zebrafish cancer: the state of the art and the path forward. 13, 624-636. 10.1038/nrc3589 PMC6040891 23969693

[DMM030056C58] Williams C. B., Yeh E. S. and Soloff A. C. (2016). Tumor-associated macrophages: unwitting accomplices in breast cancer malignancy. 2, 15025. 10.1038/npjbcancer.2015.25 PMC4794275 26998515

[DMM030056C59] Yan C., Yang Q. and Gong Z. (2017). Tumor-associated neutrophils and macrophages promote gender disparity in hepatocellular carcinoma in zebrafish. 77, 1395-1407. 10.1158/0008-5472.CAN-16-2200 28202512

[DMM030056C60] Yang G.-W., Jiang J.-S. and Lu W.-Q. (2015). Ferulic acid exerts anti-angiogenic and anti-tumor activity by targeting fibroblast growth factor receptor 1-mediated angiogenesis. 16, 24011-24031. 10.3390/ijms161024011 PMC4632735 26473837

