# Supplementary material for: Generation of a double binary transgenic zebrafish model to study myeloid gene regulation in response to oncogene activation in melanocytes
Source: Dis Model Mech. 2018 Apr 6;11(4):dmm030056. doi: 10.1242/dmm.030056 (PMC5963855; doi:10.1242/dmm.030056)
Supplement: Supplementary information [file dmm-11-030056-s1.pdf]

Table S1. dataset_melan_nutri_S1.xlsx

Click here to Download Table S1

pCrysβ:ECFP-LexOP:mCherry-NRasK61Q
pCrysβ:ECFP-LexOP:mCherry-HrasG12V
pCrysβ:ECFP-LexOP:Cherry-KrasG12V
pCR-II-TOPO-mitfa_LexPR-2A-Cerulean-SV40pA-FRT-Kan-FRT
pCR-II-TOPO-kita_LexPR-2A-Cerulean-SV40pA-FRT-Kan-FRT
pGEM BirA-2A-Citrine-SV40pA-FRT-Kan-FRT

Table S2. List of plasmids generated for the purpose of this study available from Addgene

Transgenic line annotation	Allele	Abbreviation	Description
*TgBAC(mpx:BirA-Citrine)^{ox121}	ox121	mpx:BirA-Citrine	Neutrophil BirA driver
TgBAC(mpeg1:BirA-Citrine)^{ox122}	ox122	mpeg1:BirA-Citrine	Macrophage BirA driver
TgBAC(kita:LexPR-Cerulean)^{ox123}	ox123	kita:LexPR-Cerulean	Melanocyte LexPR driver
TgBAC(mitfa:LexPR-Cerulean)^{ox124}	ox124	mitfa:LexPR-Cerulean	Melanocyte LexPR driver
Tg(Crysβ:ECFP, LexOP:mCherry-NRas^{K61Q})^{ox125}	ox125	LexOP:mCherry-NRas^{K61Q}	NRas effector
Tg(Crysβ:ECFP, LexOP:mCherry-Hras^{G12V})^{ox126}	ox126	LexOP:mCherry-HRas^{G12V}	HRas effector
Tg(Crysβ:ECFP, LexOP:mCherry-Kras^{G12V})^{ox127}	ox127	LexOP:mCherry-KRas^{G12V}	KRas effector
Tg(kita:LexPR-Cerulean;LexOP:mCherry-NRas^{K61Q})^{ox129}	ox129	kita:LexPR;LexOP:NRas^{K61Q}	NRas – kita transformed melanocytes
Tg(mitfa:LexPR-Cerulean;LexOP:mCherry-NRas^{K61Q})^{ox130}	ox130	mitfa:LexPR;LexOP:NRas^{K61Q}	NRas – mitfa transformed melanocytes
Tg(kita:LexPR-Cerulean;LexOP:mCherry-HRas^{G12V})^{ox131}	ox131	kita:LexPR;LexOP:HRas	HRas – kita transformed melanocytes
Tg(kita:LexPR-Cerulean;LexOP:mCherry-KRas^{G12V})^{ox132}	ox132	kita:LexPR;LexOP:KRas	KRas – kita transformed melanocytes
*Tg(bactin:Avi-Cerulean-Rangap)^{ct700a}	ct700a	bactin:Avi-Cerulean-Rangap	Nuclear envelope Avi-Rangap effector
TgBAC(mpx:BirA-Citrine;bactin:Avi-Cerulean-Rangap)^{ox125}	ox128	mpx:BirA;bactin:Avi-Rangap	Biotagged neutrophil nuclei
TgBAC(mpeg1:BirA-Citrine;bactin:Avi-Cerulean-Rangap)^{ox133}	ox133	mpeg1:BirA;bactin:Avi-Rangap	Biotagged macrophage nuclei

Table S3. Zebrafish transgenic lines used in this study. Two previously generated lines are marked with an asterisk.

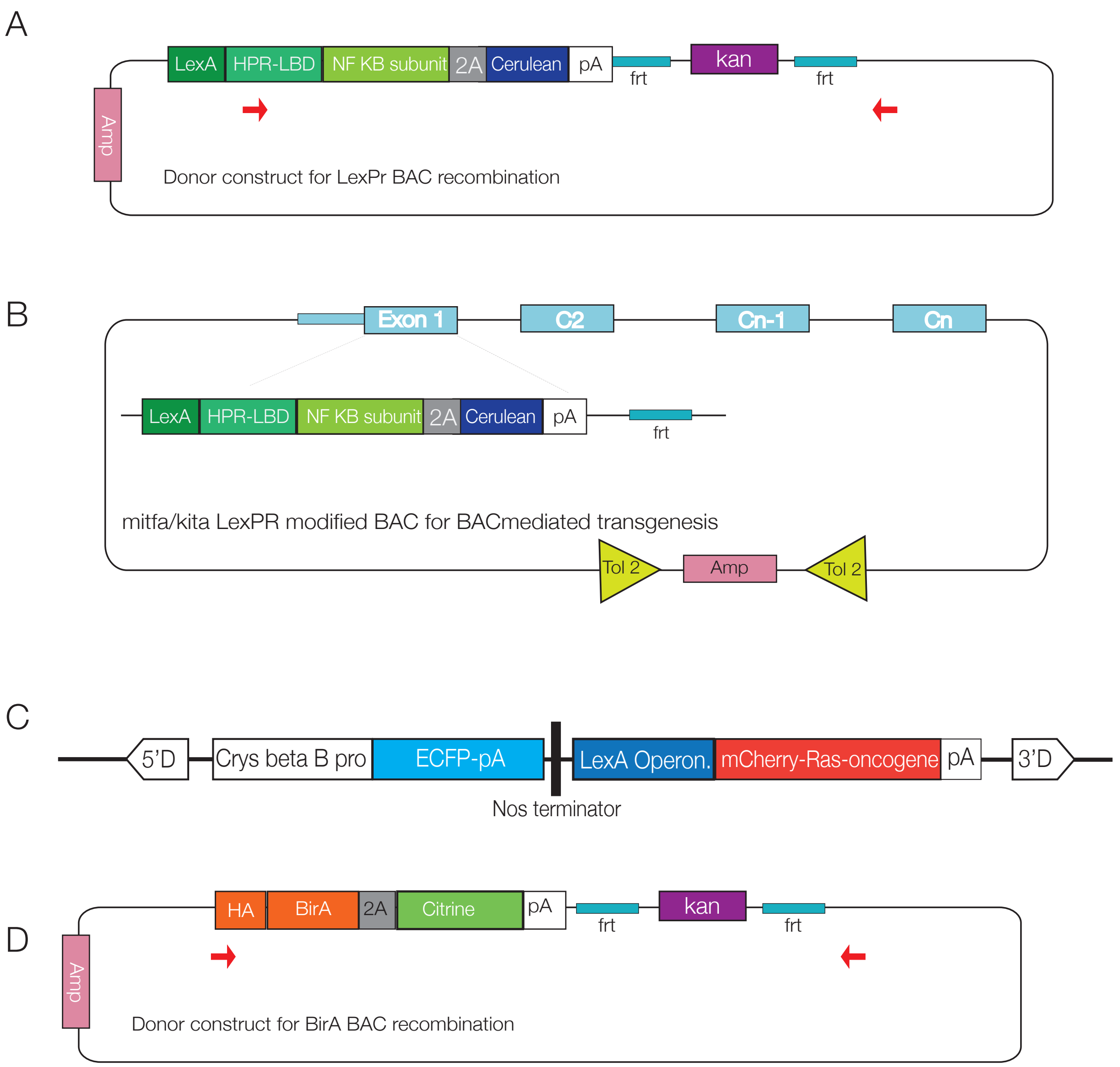

Previously used for generation of mpo BirA driver line - Kenyon et. al.

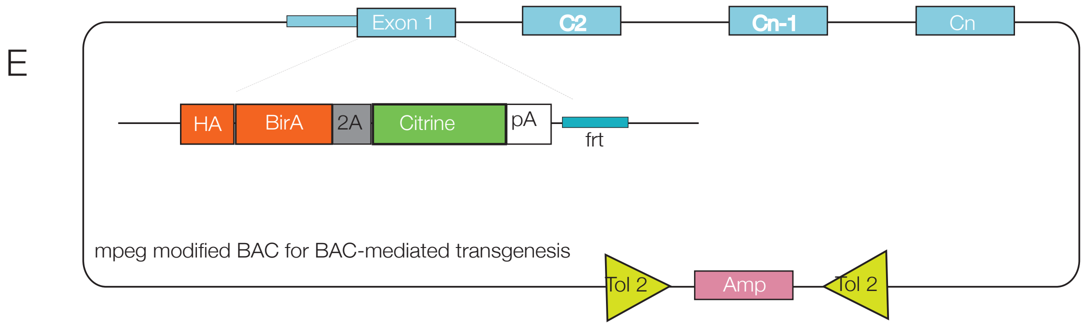

Disease Models & Mechanisms • Supplementary information

Figure S1. Constructs used in generation of model system. (A) Schematic of the mpx BAC donor construct, containing HA-tagged BirA (orange), a ribosomal skipping motif - 2A (grey), citrine reporter (green), polyA tail (white), followed by FRT recombination sites (turquoise) flanking a kanamycin selection cassette. Ampicillin selection cassette, not amplified as a part of recombination cassette, is used as a selection marker for E. coli during plasmid DNA isolation. Red arrows indicate position of primers used for amplification and recombination into the mpx BAC. (B) Schematic of kita/mitfa modified BAC DNA containing the LexPR transactivator (green), a viral self-cleaving peptide (2A, grey) and a fluorescent reporter (cerulean, blue), followed by a polyA tail (pA, white) and remaining Frt site, recombined into the first exon, with an ampicillin selection cassette (Amp, pink) and tol2 arms (yellow) on the BAC plasmid backbone for BAC mediated transgenesis. (C) Schematic PCrysB:ECFP-LexOP-mCherry-RasOncogene construct, embedded within a non-autonomous Ds element to produce Ds insertions in the zebrafish genome with the aid of a modified Ac transposase. (D) Schematic of the mpeg1 BAC donor construct, containing HA-tagged BirA (orange), a ribosomal skipping motif - 2A (grey), citrine reporter (green), polyA tail (white), followed by FRT recombination sites (turquoise) flanking a kanamycin selection cassette. Ampicillin selection cassette, not amplified as a part of recombination cassette, is used as a selection marker for E. coli during plasmid DNA isolation. Red arrows indicate position of primers used for amplification and recombination into the mpeg1 BAC. (E) Schematic of mpeg1 modified BAC DNA with HA-tagged BirA (orange), a ribosomal skipping motif - 2A (grey), citrine reporter (green), polyA tail (white), followed by the remaining FRT sites (turquoise) recombined into the first exon, with a BAC-specific ampicillin-Tol2 cassette (iTol2) in pink and yellow.

A

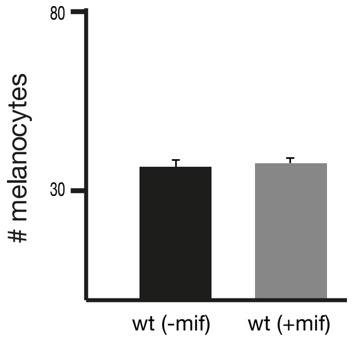

Figure S2. Comparison of melanocyte number in wildtype larvae with and without mifepristone. (A). Graph showing no change in cranial melanocyte number in wildtype larvae at 5 dpf (n=7).

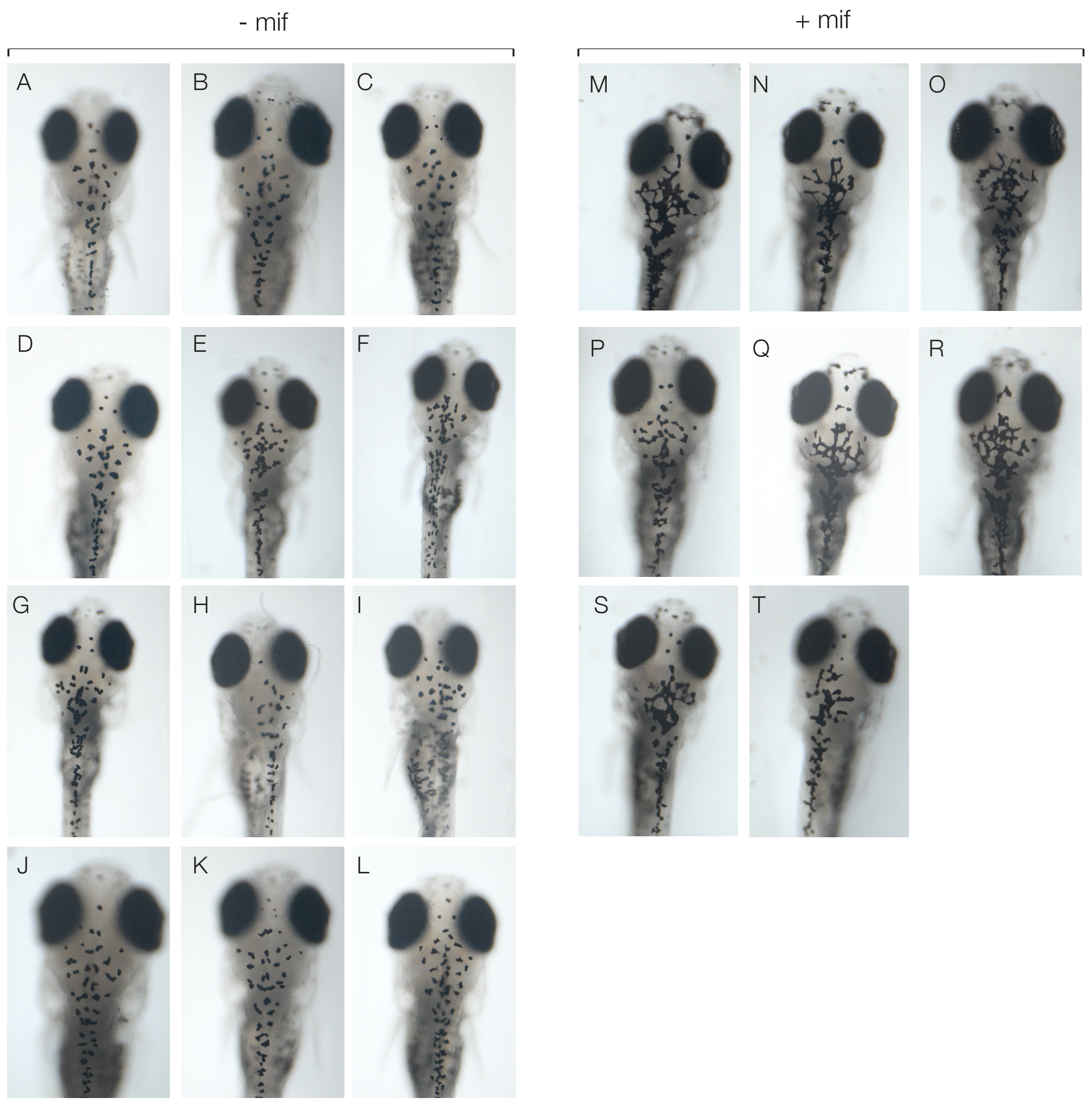

Figure S3. Comparison of melanocyte morphology in *Tg(kita:LexPR-Cerulean;LexOP:mCherry-NRas)*^{ox12} in oncogene activated (+mif) 12 dpf larvae versus controls (-mif). (A-T) Dorsal views of the head of 12 dpf larvae without (A-L) and with mifepristone-dependent mCherry-NRasQ61K activation (red) in *Tg(kita:LexPR-Cerulean;LexOP:mCherry-NRas)*^{ox12} (M-T).

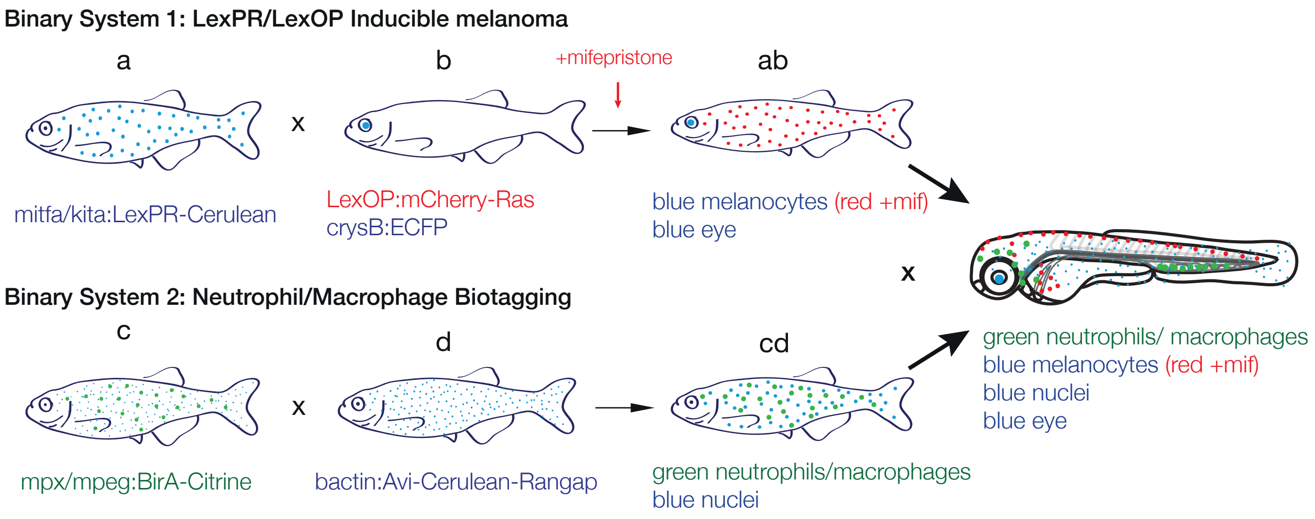

Figure S4. Double binary system to study the myeloid response to oncogene-activated melanocytes. (A) Binary System 1: The LexPR transactivator expressed under the control of a melanocyte-specific promoter activates in trans the mCherry-Ras oncogene in the presence of a specific ligand (mifepristone). Fish a) Melanocyte-specific LexPR transactivator with Cerulean reporter. Fish b) Lex Operon controlled mCherry-Ras with crystallin-specific ECFP expression. **Binary System 2:** Nuclear envelope protein tagged with a biotin acceptor peptide (Avi-tag) is biotinylated in macrophages/neutrophils allowing for cell-specific isolation of nuclei and genome-wide analysis. Fish c) Macrophage/neutrophil-specific biotin ligase, BirA, with Citrine reporter. Fish d. Ubiquitous Avi-tagged nuclear envelope protein with Cerulean reporter. Fish **a** crossed to Fish **b** results in Fish **ab** (blue eyes and blue melanocytes, red in the presence of mifepristone). Fish **c** crossed to Fish **d** in Fish **cd** (green macrophages/neutrophils and blue nuclei). Fish **ab** crossed to Fish **cd** yields embryos to be analysed (green neutrophils/macrophages, blue melanocytes or red in the presence of mifepristone, blue nuclei, blue eyes).

Disease Models & Mechanisms • Supplementary information

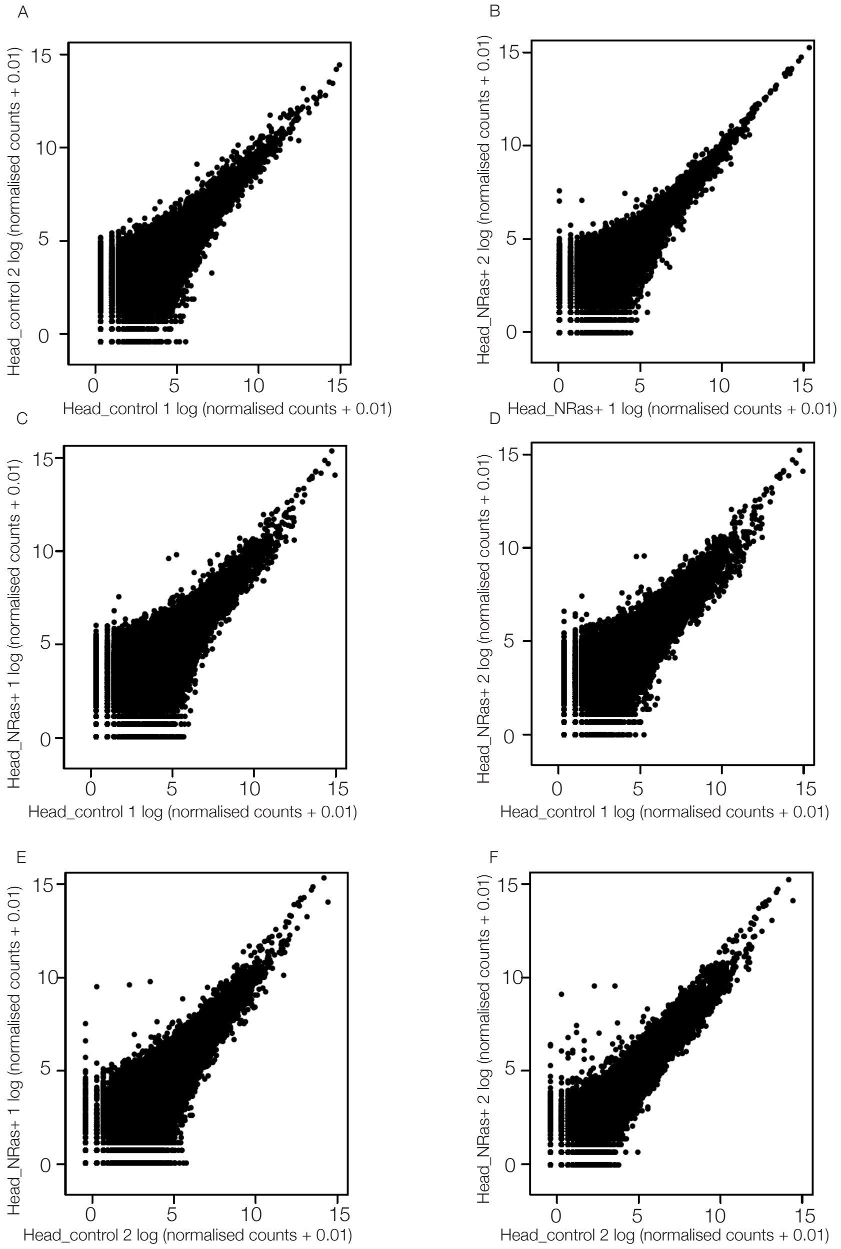

Figure S5. Technical reproducibility of replicates. (A-F) Scatter plot of log (normalised counts + 0.01) between biological duplicates for neutrophil nuclear samples head_control 2 vs head_control 1 (A), head_Nras+ 2 vs head_Nras+1 (B) head_Nras+ 1 vs head_control 1 (C), head_Nras+ 2 vs head_control 1 (D) head_Nras+ 1 vs head_control 2 (E), head_Nras+ 2 vs head_control 2 (F).

Disease Models & Mechanisms • Supplementary information

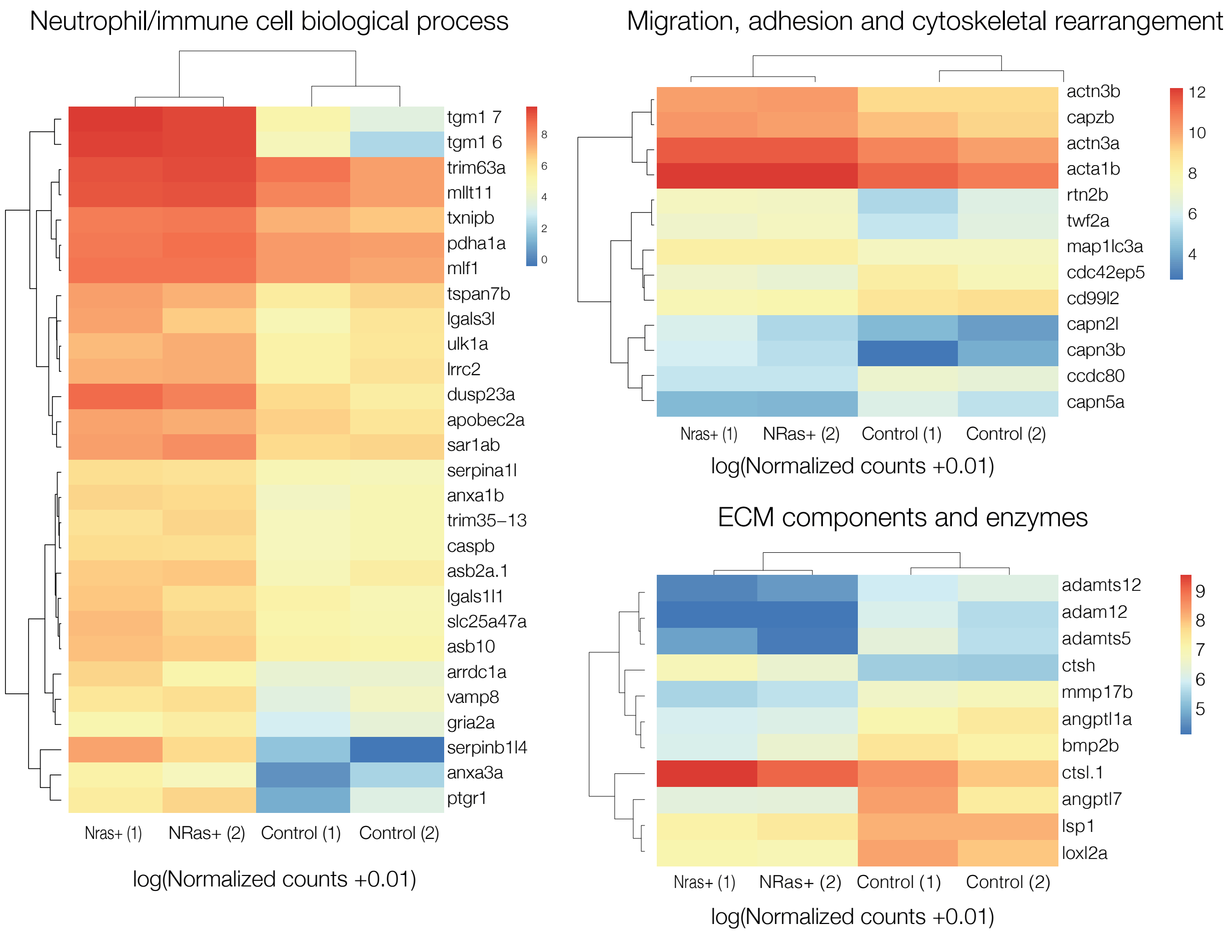

Figure S6. Classification of differentially expressed transcripts. (A-C) Heatmaps show the $\log_{10}$ (normalised counts (NMCT) +0.01) of selected differentially expressed transcripts (adjusted p-value < 0.05). Red - high expression. Yellow - medium expression. Blue - low expression. Neutrophil/immune cell biological processes (A), Migration, adhesion and cytoskeletal rearrangement (B) and ECM components and enzymes (C).

Disease Models & Mechanisms • Supplementary information

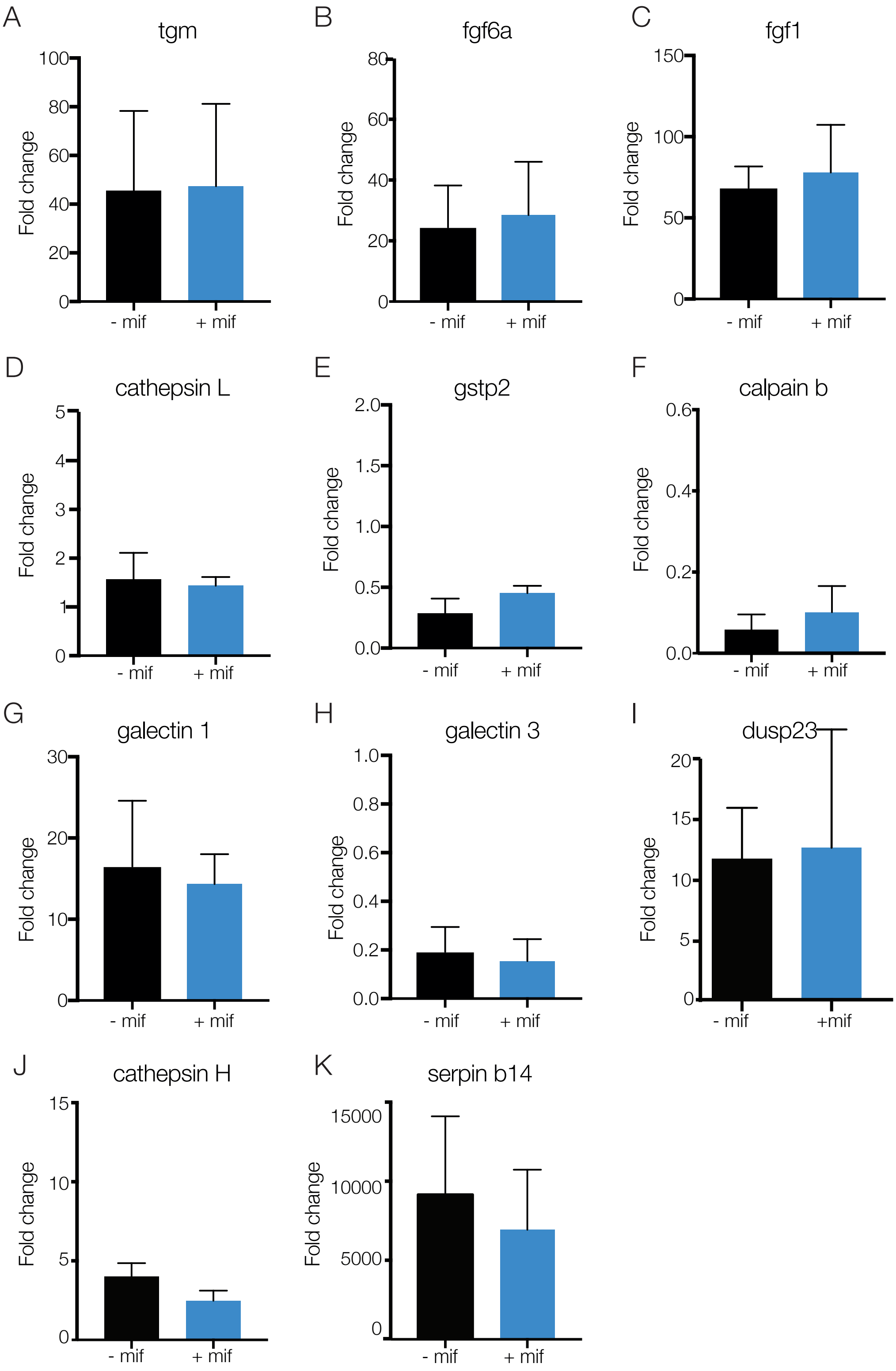

Disease Models & Mechanisms • Supplementary information

Figure S7. Analysis of the effect of mifepristone on mRNA levels in neutrophils. (A-G). Bar graphs for biological triplicates reflect little or no change in mRNA levels of selected transcripts as measured by qPCR. mpx:BirA;bactin:Avi-Rangap were reared in the absence and presence of mifepristone and nuclei pulldowns carried out at 5 dpf. Transcript levels were measured for *tgm*1 (A), fgf6a (B), fgf1 (C), cathepsin L (D), gstp2 (E), calpain b (F), galectin 1 (G), galectin 3 (H), dusp23 (I), cathepsin H (J) and serpinb14 (K) by qPCR.
